# Functional Comparison of Bacteria from the Human Gut and Closely Related Non-Gut Bacteria Reveals the Importance of Conjugation and a Paucity of Motility and Chemotaxis Functions in the Gut Environment

**DOI:** 10.1371/journal.pone.0159030

**Published:** 2016-07-14

**Authors:** Dragana Dobrijevic, Anne-Laure Abraham, Alexandre Jamet, Emmanuelle Maguin, Maarten van de Guchte

**Affiliations:** Micalis Institute, INRA, AgroParisTech, Université Paris-Saclay, 78350, Jouy-en-Josas, France; University of Palermo, ITALY

## Abstract

The human GI tract is a complex and still poorly understood environment, inhabited by one of the densest microbial communities on earth. The gut microbiota is shaped by millennia of evolution to co-exist with the host in commensal or symbiotic relationships. Members of the gut microbiota perform specific molecular functions important in the human gut environment. This can be illustrated by the presence of a highly expanded repertoire of proteins involved in carbohydrate metabolism, in phase with the large diversity of polysaccharides originating from the diet or from the host itself that can be encountered in this environment. In order to identify other bacterial functions that are important in the human gut environment, we investigated the distribution of functional groups of proteins in a group of human gut bacteria and their close non-gut relatives. Complementary to earlier global comparisons between different ecosystems, this approach should allow a closer focus on a group of functions directly related to the gut environment while avoiding functions related to taxonomically divergent microbiota composition, which may or may not be relevant for gut homeostasis. We identified several functions that are overrepresented in the human gut bacteria which had not been recognized in a global approach. The observed under-representation of certain other functions may be equally important for gut homeostasis. Together, these analyses provide us with new information about this environment so critical to our health and well-being.

## Introduction

The human gastrointestinal system, and especially the distal gut, is inhabited by one of the densest populations of microorganisms known. The importance of this community, the human gut microbiota, for human health and wellbeing is now well documented. While some of the cultivable bacteria living in the human gut have been studied for decades (for instance *Escherichia coli*), the development of new DNA sequencing technologies and the concept of metagenomics provided a paradigm-changing shift in the study of the human gut microbiota. The collective genetic information of the human gut microbiota, the human gut metagenome, is currently the focus of intense international sequencing and research efforts. A first catalogue of 3.3 million human gut microbial genes has been established in 2010 [1], and more recently an extensive update of this catalogue was published, combining data from different sources and containing nearly 10 million genes [2]. Apart from metagenomics projects, data derive from sequencing efforts targeting the genomes of specific bacteria from the human gut. So far, sequences of 778 gut-associated bacterial genomes are available through the Human Microbiome Project [3], thus giving access to an independent complementary line of investigation of the human gut microbiota.

More than 90% of gut bacteria are members of only two phyla, *Bacteroidetes* and *Firmicutes*, the relative proportions of which exhibit a continuous gradient within the human population [4, 5]. Within the borders of these phyla the microbiota composition is highly individual-specific, showing high variability at the species and strain levels. In contrast to this taxonomic diversity however, functional profiles are far less variable across healthy individuals [1, 6, 7], confirming the existence of a well-balanced host-microbial symbiosis.

The results of functional analyses of the human gut metagenome showed that the proteome of the human gut microbiota is enriched in proteins involved in carbohydrate metabolism, energy metabolism and storage, generation of short-chain fatty acids, amino acids metabolism, biosynthesis of secondary metabolites and metabolism of cofactors and vitamins [1, 4, 6–8]. In particular, the human gut microbiota provides a broad and diverse array of carbohydrate-active enzymes, many of which are not present in the human glycobiome [9]. Similar observations were made during genome analysis of some of the human gut-associated bacteria, such as *Bacteroides fragilis*, *Bacteriodes thetaiotaomicron* or *Methanobrevibacter smithii* [10, 11]. Additionally, many yet uncharacterized or completely novel protein families were shown to be specific to the human gut, suggesting that many unknown and uncharacterized processes are yet to be discovered in this environment [12].

The formentioned catalogue containing 3.3 million non-redundant microbial genes from the intestinal tract of 124 individuals [1] provided an opportunity to differentiate bacterial functions necessary for a bacterium to thrive in the gut environment, and therefore present in every gut bacterial species, from those involved in the homeostasis of the whole gut ecosystem, encoded across many bacterial species. Qin *et al*. referred to these functions as (bacterial) "minimal gut genome" and "minimal gut metagenome", respectively [1]. The minimal gut metagenome includes functions known to be important to the host—bacterial interaction, such as the capacities to metabolize complex polysaccharides and to synthesize short-chain fatty acids, indispensable amino acids and vitamins. It also includes a considerable fraction of functions (~45%) that were present in less than 10% of earlier sequenced, mainly non-gut, bacterial genomes. These "otherwise rare", gut-specific, functions mainly contained uncharacterized genes, underscoring our limited knowledge of gut functioning.

Here we present a different approach to the identification of functions that may inform us on the conditions of gut homeostasis, comparing the predicted proteomes of fully sequenced gut bacteria to those of closely related bacteria from other environments. The underlying hypothesis is that the comparison of metagenomic data from different ecosystems, for example gut and soil, may reveal functions that are characteristic of either ecosystem partly because of the different constraints of each system (i.e. adaptive functions) and partly because the two ecosystems are populated by different bacterial phyla with inherent differences between them (which may or may not be important in adaptation to the environment). Also, the global approach would not recognize the importance of certain functions in the gut environment if these functions also play a role in (other bacteria in) other environments. The comparison of closely related bacterial species from different environments allows focusing on differences that are directly related to the different environments.

## Materials and Methods

### Data preparation

Information on sequenced genomes was downloaded from the GOLD database (www.genomesonline.org) (version of 07 March 2013). Gene and protein sequences were downloaded from NCBI via iMOMi [13] except for those of *Megamonas rupellensis* DSM 1994 and *Caloramator australicus* KCTC 5601 which were downloaded from Integrated Microbial Genomes data warehouse (www.img.jgi.doe.gov).

### Phylogenetic analysis

The 16S rRNA gene sequences used in this study were retrieved in fasta format from GenBank (NCBI-GenBank Flat File Release 195.0). Phylogenetic analysis was conducted using the SeaView 4.4.1 platform [14]. MUSCLE [15] was used for multiple sequence alignment with default parameters and blocks of evolutionary conserved sites were selected by Gblocks [16]. The tree was computed using phyML 3.0 [16] based on the Maximum Likelihood method and visualized using iTOL v3.0 [17]. The 16S rRNA gene sequence of *Methanobrevibacter smithii* ATCC 35061 was used as an outgroup for the analysis.

### Protein functional annotation and localization prediction

Protein functional annotations were made by BLASTP search against the eggNOGv3.0 bactNOG catalog [18] and the best hit (e-value <10e-5) was retained. To predict protein localization, protein sequences were analyzed using SurfG+ [19]. Functional comparisons were performed using R (https://www.r-project.org): functional clustering of the bacterial species used in this study was performed with the heatmap function taking bactNOGs present in ≥ 2 species and ≤ 45 species into account (i.e. bactNOGs present in only one species or in all 46 species studied, which are not informative for clustering, were ignored); the distribution of functions among gut and non-gut genomes was visualized with the function hist2d of the gplots library (version 2.17.0), bin contents was summarized with a log function.

## Results

### Closely related gut and non-gut bacterial species constitute distinct functional groups

**Fig 1:**
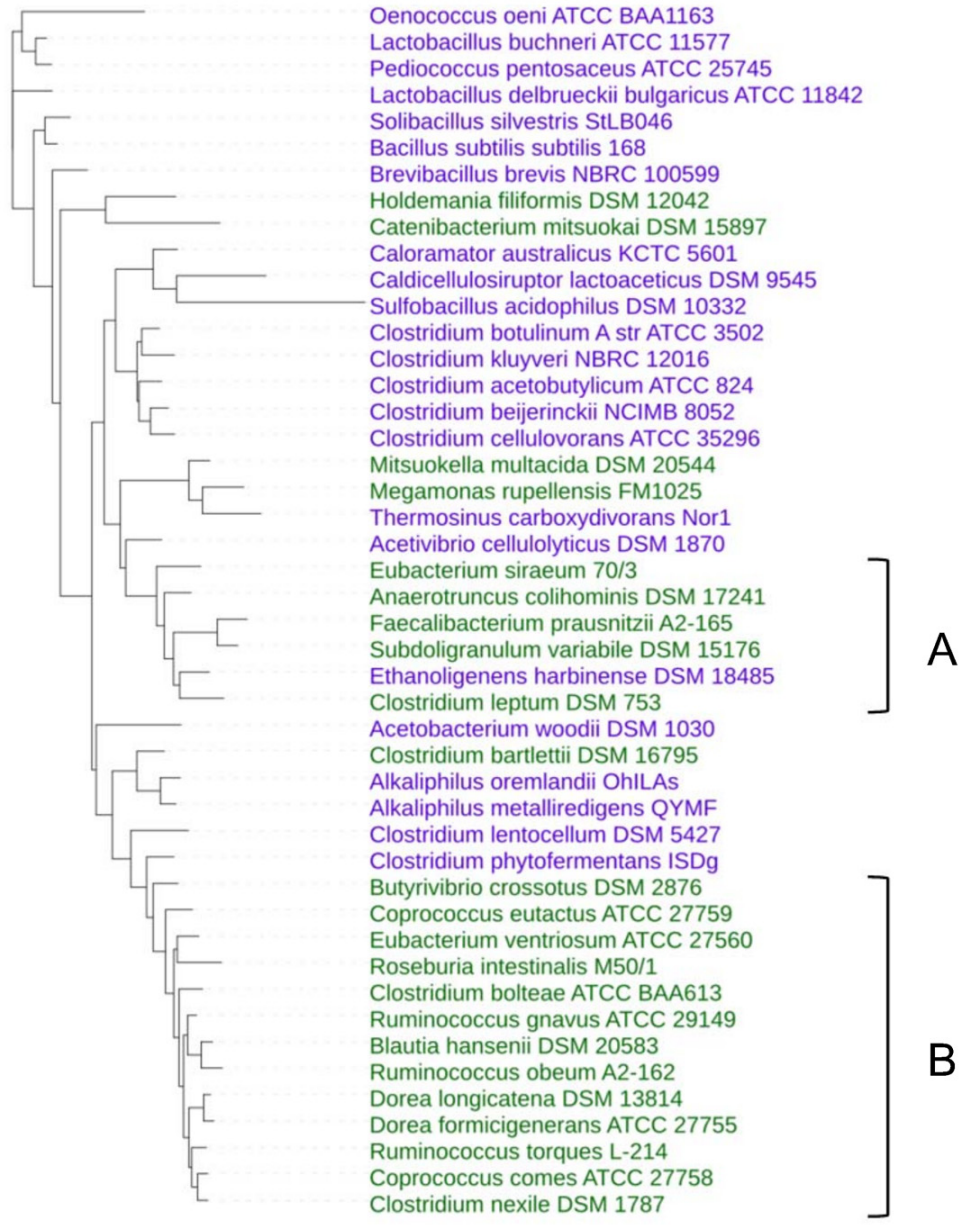
Phylogeny of bacterial species used in this study. 16S rRNA based tree by Maximum Likelihood method. Green, gut species; Violet, non-gut species. Clusters A and B make part of one functional cluster (Fig 2).

Bacterial diversity in the gut is largely restricted to two major phyla, *Firmicutes* and *Bacteroidetes*. In this study, we chose to focus on gut bacteria from the *Firmicutes* phylum and compare the functions encoded in their genomes with those encoded in closely related bacteria from other environments. Information on sequenced genomes was downloaded from the GOLD database (www.genomesonline.org) and filtered to select publicly available complete genomes from the *Firmicutes* phylum. Among these, "gut species" were identified by comparison with the gut metagenome data presented in [1], choosing species that in the latter study were marked "frequent" (i.e. highly abundant in the gut) and/or "common" (i.e. present in the gut of many individuals). "Non-gut" species, for which no association with the gut environment could be found in literature, were chosen in taxonomic groups as close as possible to the gut-species. To validate this choice, the gene repertoires of the selected species (one strain per species) were analyzed using BLASTn against the MetaHIT gut microbiota gene catalog [1] to identify genes present in this catalog (sequence identity ≥ 95% over ≥ 90% of the longest sequence length). A species was considered as “gut species” if at least 5% of the genes were present in the catalog, and as “non-gut” species if less than 0.5% of the genes were present in the catalog. This resulted in the constitution of two genome sets with “gut” or “non-gut” attributions, respectively, that were coherent between data obtained from literature and from metagenomic sequencing of fecal samples. The first set represents 23 bacterial species isolated from human stool samples. The second set represents 23 closely related species from other environments (Table 1, Fig 1). We refer to these sets of genomes as “gut” and “non-gut”, respectively. The majority of the selected species belongs to the class *Clostridia*, order *Clostridiales* (19 gut species and 14 non-gut species).

**Table 1:** Bacterial genomes used in this study.

Bacteria	Environment		Class	Order	Family
Bacillus subtilis 168	non gut	soil	Bacilli	Bacillales	Bacillaceae
Brevibacillus brevis NBRC 100599	non gut	soil	Bacilli	Bacillales	Paenibacillaceae
Solibacillus silvestris StLB046	non gut	soil	Bacilli	Bacillales	Planococcaceae
Lactobacillus buchneri ATCC 11577	non gut	silage	Bacilli	Lactobacillales	Lactobacillaceae
Lactobacillus delbrueckii bulgaricus ATCC 11842	non gut	yoghurt	Bacilli	Lactobacillales	Lactobacillaceae
Pediococcus pentosaceus ATCC 25745	non gut	plants, cheese	Bacilli	Lactobacillales	Lactobacillaceae
Oenococcus oeni ATCC BAA 1163	non gut	wine	Bacilli	Lactobacillales	Leuconostocaceae
Alkaliphilus metalliredigens QYMF	non gut	borax leachate ponds	Clostridia	Clostridiales	Clostridiaceae
Alkaliphilus oremlandii OhILAs	non gut		Clostridia	Clostridiales	Clostridiaceae
Caloramator australicus KCTC 5601	non gut		Clostridia	Clostridiales	Clostridiaceae
Clostridium acetobutylicum ATCC 824	non gut		Clostridia	Clostridiales	Clostridiaceae
Clostridium beijerinckii NCIMB 8052	non gut	soil	Clostridia	Clostridiales	Clostridiaceae
Clostridium botulinum A str ATCC 3502	non gut		Clostridia	Clostridiales	Clostridiaceae
Clostridium cellulovorans ATCC 35296	non gut		Clostridia	Clostridiales	Clostridiaceae
Clostridium kluyveri NBRC 12016	non gut		Clostridia	Clostridiales	Clostridiaceae
Sulfobacillus acidophilus DSM 10332	non gut	geothermal environments, mines	Clostridia	Clostridiales	Clostridiales Family XVII
Acetobacterium woodii DSM 1030	non gut		Clostridia	Clostridiales	Eubacteriaceae
Eubacterium ventriosum ATCC 27560	gut		Clostridia	Clostridiales	Eubacteriaceae
Blautia hansenii DSM 20583	gut		Clostridia	Clostridiales	Lachnospiraceae
Butyrivibrio crossotus DSM 2876	gut		Clostridia	Clostridiales	Lachnospiraceae
Clostridium bolteae ATCC BAA 613	gut		Clostridia	Clostridiales	Lachnospiraceae
Clostridium lentocellum DSM 5427	non gut		Clostridia	Clostridiales	Lachnospiraceae
Clostridium nexile DSM 1787	gut		Clostridia	Clostridiales	Lachnospiraceae
Clostridium phytofermentans ISDg	non gut		Clostridia	Clostridiales	Lachnospiraceae
Coprococcus comes ATCC 27758	gut		Clostridia	Clostridiales	Lachnospiraceae
Coprococcus eutactus ATCC 27759	gut		Clostridia	Clostridiales	Lachnospiraceae
Dorea formicigenerans ATCC 27755	gut		Clostridia	Clostridiales	Lachnospiraceae
Dorea longicatena DSM 13814	gut		Clostridia	Clostridiales	Lachnospiraceae
Roseburia intestinalis M50/1	gut		Clostridia	Clostridiales	Lachnospiraceae
Ruminococcus gnavus ATCC 29149	gut		Clostridia	Clostridiales	Lachnospiraceae
Ruminococcus obeum A2-162	gut		Clostridia	Clostridiales	Lachnospiraceae
Ruminococcus torques L2-14	gut		Clostridia	Clostridiales	Lachnospiraceae
Clostridium bartlettii DSM 16795	gut		Clostridia	Clostridiales	Peptostreptococcaceae
Acetivibrio cellulolyticus CD2	non gut	sewage sludge	Clostridia	Clostridiales	Ruminococcaceae
Anaerotruncus colihominis DSM 17241	gut		Clostridia	Clostridiales	Ruminococcaceae
Clostridium leptum DSM 753	gut		Clostridia	Clostridiales	Ruminococcaceae
Ethanoligenens harbinense DSM 18485	non gut	anaerobic activated sludge of molasses wastewater	Clostridia	Clostridiales	Ruminococcaceae
Eubacterium siraeum 70/3	gut		Clostridia	Clostridiales	Ruminococcaceae
Faecalibacterium prausnitzii A2-165	gut		Clostridia	Clostridiales	Ruminococcaceae
Subdoligranulum variabile DSM 15176	gut		Clostridia	Clostridiales	Ruminococcaceae
Caldicellulosiruptor lactoaceticus DSM 9545	non gut		Clostridia	Thermoanaerobacterales	Thermoanaerobacterales Family III.
Catenibacterium mitsuokai DSM 15897	gut		Erysipelotrichia	Erysipelotrichales	Erysipelotrichaceae
Holdemania filiformis DSM 12042	gut		Erysipelotrichia	Erysipelotrichales	Erysipelotrichaceae
Megamonas rupellensis DSM 19944	gut		Negativicutes	Selenomonadales	Veillonellaceae
Mitsuokella multacida DSM 20544	gut		Negativicutes	Selenomonadales	Veillonellaceae
Thermosinus carboxydivorans Nor1	non gut	hot spring	Negativicutes	Selenomonadales	Veillonellaceae

All bacteria belong to the *Firmicutes* phylum, the sequencing status of their respective genomes is "complete" and sequences are publicly available (GOLD database: http://www.genomesonline.org/cgi-bin/GOLD/index.cgi).

**Fig 2:**
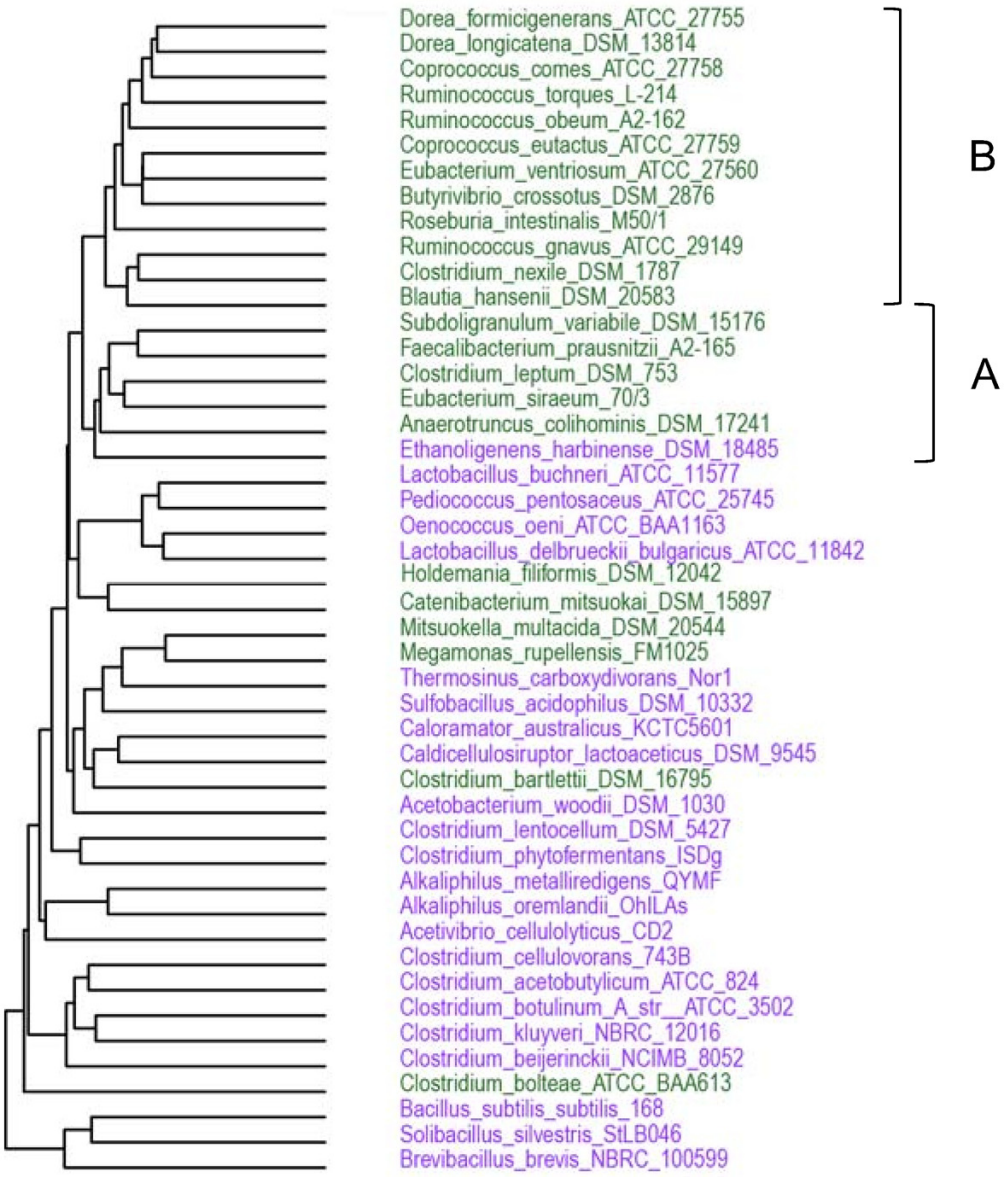
Functional clustering of bacterial species used in this study. Clustering of 46 species on the basis of functional profiles (presence or absence of bactNOGs). Green, gut species; Violet, non-gut species. A and B represent phylogenetically distinct clusters (Fig 1).

To evaluate the functional composition of the species studied, we attributed the predicted protein complement of each genome to orthologous groups (bactNOGs) using the eggNOG v.3.0 database [18]. Orthologous groups of proteins have proven useful for functional analyses, as orthologs tend to have equivalent functions [20]. The fraction of the proteins that could be attributed to bactNOGs varied from 48 to 81% depending on the species (Table 2), and was generally higher for non-gut species than for gut species (71% vs 60%, respectively, on an average). For each genome this resulted in a functional profile based on the presence of bactNOGs, which was subsequently used as a basis for functional genome clustering (Fig 2). With only few exceptions, the gut bacterial species studied appear to form a distinct cluster. Notably, the phylogenetically distinct gut bacteria clusters A and B (Fig 1) appear to be united in one functional cluster (Fig 2).

**Table 2:** Protein functional annotation and localization prediction per genome.

Bacteria	PSE	SEC	CYTO	MEMB	Total	eggNOG	eggNOG %
Anaerotruncus colihominis DSM 17241	304	111	3496	516	4427	2214	50,0
Blautia hansenii DSM 20583	292	80	2326	473	3171	2029	64,0
Butyrivibrio crossotus DSM 2876	291	92	1803	343	2529	1669	66,0
Catenibacterium mitsuokai DSM 15897	211	69	2311	386	2977	1799	60,4
Clostridium bartlettii DSM 16795	245	88	2025	429	2787	2001	71,8
Clostridium bolteae ATCC BAA 613	644	188	5386	1066	7284	3473	47,7
Clostridium leptum DSM 753	293	91	3055	484	3923	1930	49,2
Clostridium nexile DSM 1787	423	85	3152	579	4239	2157	50,9
Coprococcus comes ATCC 27758	303	69	2954	587	3913	2055	52,5
Coprococcus eutactus ATCC 27759	320	84	2166	412	2982	1859	62,3
Dorea formicigenerans ATCC 27755	301	65	2438	473	3277	2072	63,2
Dorea longicatena DSM 13814	248	53	2234	435	2970	1930	65,0
Eubacterium siraeum 70/3	252	85	1702	308	2347	1486	63,3
Eubacterium ventriosum ATCC 27560	255	168	1953	426	2802	1721	61,4
Faecalibacterium prausnitzii A2-165	250	118	2644	463	3475	1849	53,2
Holdemania filiformis DSM 12042	416	110	3075	622	4223	2167	51,3
Megamonas rupellensis DSM 19944	137	130	1597	361	2225	1729	77,7
Mitsuokella multacida DSM 20544	162	145	1885	366	2558	1768	69,1
Roseburia intestinalis M50/1	327	114	2547	490	3478	2149	61,8
Ruminococcus gnavus ATCC 29149	339	73	2958	543	3913	2266	57,9
Ruminococcus obeum A2-162	279	177	2255	444	3155	2015	63,9
Ruminococcus torques L2-14	248	55	2108	387	2798	1902	68,0
Subdoligranulum variabile DSM 15176	319	90	2487	485	3381	2013	59,5
gut mean	298	102	2546	482	3428	2011	60
gut SEM	21	8	167	31	218	78	2
Acetivibrio cellulolyticus CD2	591	435	3313	609	4948	2882	58,2
Acetobacterium woodii DSM 1030	329	84	2611	449	3474	2424	69,8
Alkaliphilus metalliredigens QYMF	506	113	3284	722	4625	3154	68,2
Alkaliphilus oremlandii OhILAs	327	102	1972	435	2836	2250	79,3
Bacillus subtilis subtilis 168	324	210	2867	775	4177	3035	72,7
Brevibacillus brevis NBRC 100599	585	333	4139	890	5947	3932	66,1
Caldicellulosiruptor lactoaceticus DSM 9545	194	144	1692	288	2319	1807	77,9
Caloramator australicus KCTC 5601	205	87	2051	382	2725	1991	73,1
Clostridium acetobutylicum ATCC 824	399	194	2702	552	3847	2790	72,5
Clostridium beijerinckii NCIMB 8052	496	228	3594	702	5021	3416	68,0
Clostridium botulinum A str ATCC 3502	319	128	2561	582	3591	2622	73,0
Clostridium cellulovorans ATCC 35296	459	254	2995	546	4255	2882	67,7
Clostridium kluyveri NBRC 12016	358	94	2572	499	3523	2584	73,3
Clostridium lentocellum DSM 5427	473	204	2887	618	4183	2792	66,7
Clostridium phytofermentans ISDg	538	141	2628	595	3903	2833	72,6
Ethanoligenens harbinense DSM 18485	243	94	1983	381	2702	1963	72,6
Lactobacillus buchneri ATCC 11577	183	172	2118	529	3002	1886	62,8
Lactobacillus delbrueckii bulgaricus ATCC 11842	116	70	1099	244	1530	1125	73,5
Oenococcus oeni ATCC BAA 1163	83	64	986	265	1675	1130	67,5
Pediococcus pentosaceus ATCC 25745	134	42	1282	297	1755	1418	80,8
Solibacillus silvestris StLB046	402	132	2621	668	3823	2705	70,8
Sulfobacillus acidophilus DSM 10332	258	108	2522	583	3471	2172	62,6
Thermosinus carboxydivorans Nor1	194	144	2006	406	2750	2087	75,9
non-gut mean	335	156	2456	522	3482	2430	71
non-gut SEM	32	19	162	36	234	146	1

PSE, SEC, CYT, MEMB, predicted numbers of potentially surface exposed, secreted, cytoplasmic and membrane proteins, respectively, per genome. Total, total number of proteins encoded per genome. eggNOG and eggNOG %, number and % of proteins, respectively, assigned to bactNOGs in the eggNOG v.3.0 database. Gut bacteria are grouped in the upper part of the table, non-gut bacteria in the lower part.

### Functional adaptation to the human gut environment through gut-specific functions

**Fig 3:**
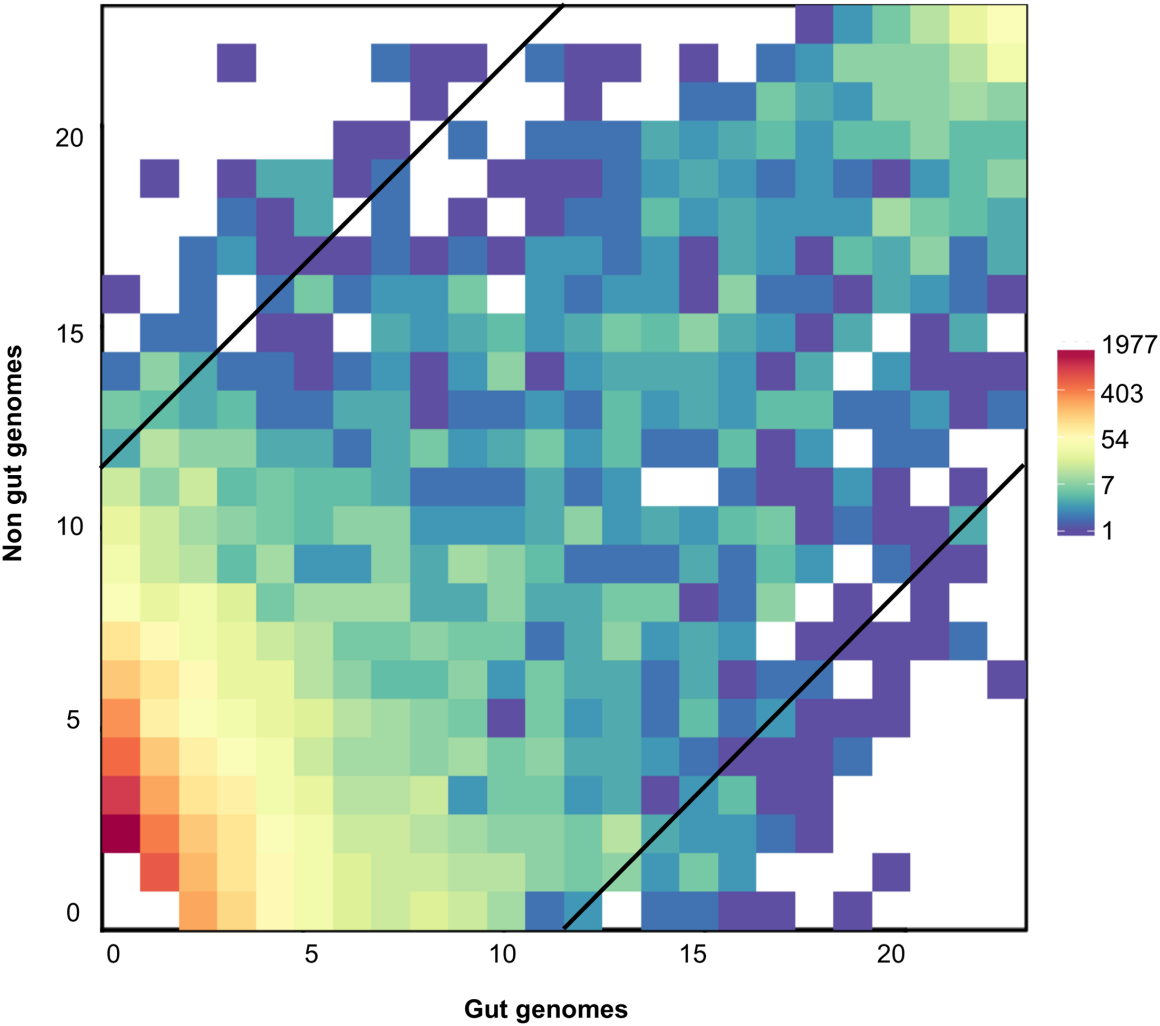
Distribution of functions among gut and non-gut genomes. Squares indicate bactNOGs as a function of the number of gut genomes (horizontal axis) and the number of non-gut genomes (vertical axis) in which they are encoded. The colours of the squares indicate the numbers of different bactNOGs at each position. BactNOGs encoded in only one of the 46 genomes are not indicated. Diagonal lines separate bactNOGs that are overrepresented in the gut genomes (bottom right), bactNOGs that are underrepresented in the gut genomes (top left), and bactNOGs with an intermediate position (see text for details).

In order to examine potential gut adaptation among *Firmicutes*, we compared the distribution of bactNOGs among gut and non-gut bacteria (Fig 3). The majority of the 20,426 detected bactNOGs was present in only one of the 46 bacterial species studied (7924 bactNOGs, not shown), or shared between two (3527 bactNOGs) or three species (2164 bactNOGs) (Fig 3, bottom left). For further analysis, bactNOGs for which the number of gut genomes where the bactNOG is represented exceeds the number of non-gut genomes where the bactNOG is represented by at least 12 were regarded as “overrepresented” in genomes of gut *Firmicutes*. This means that the representation of functions that are regarded as overrepresented varies from "present in at least 12 of the 23 gut genomes and none of the 23 non-gut genomes" to "present in all 23 gut genomes and 11 or less of the 23 non-gut genomes" (Fig 3).

**Fig 4:**
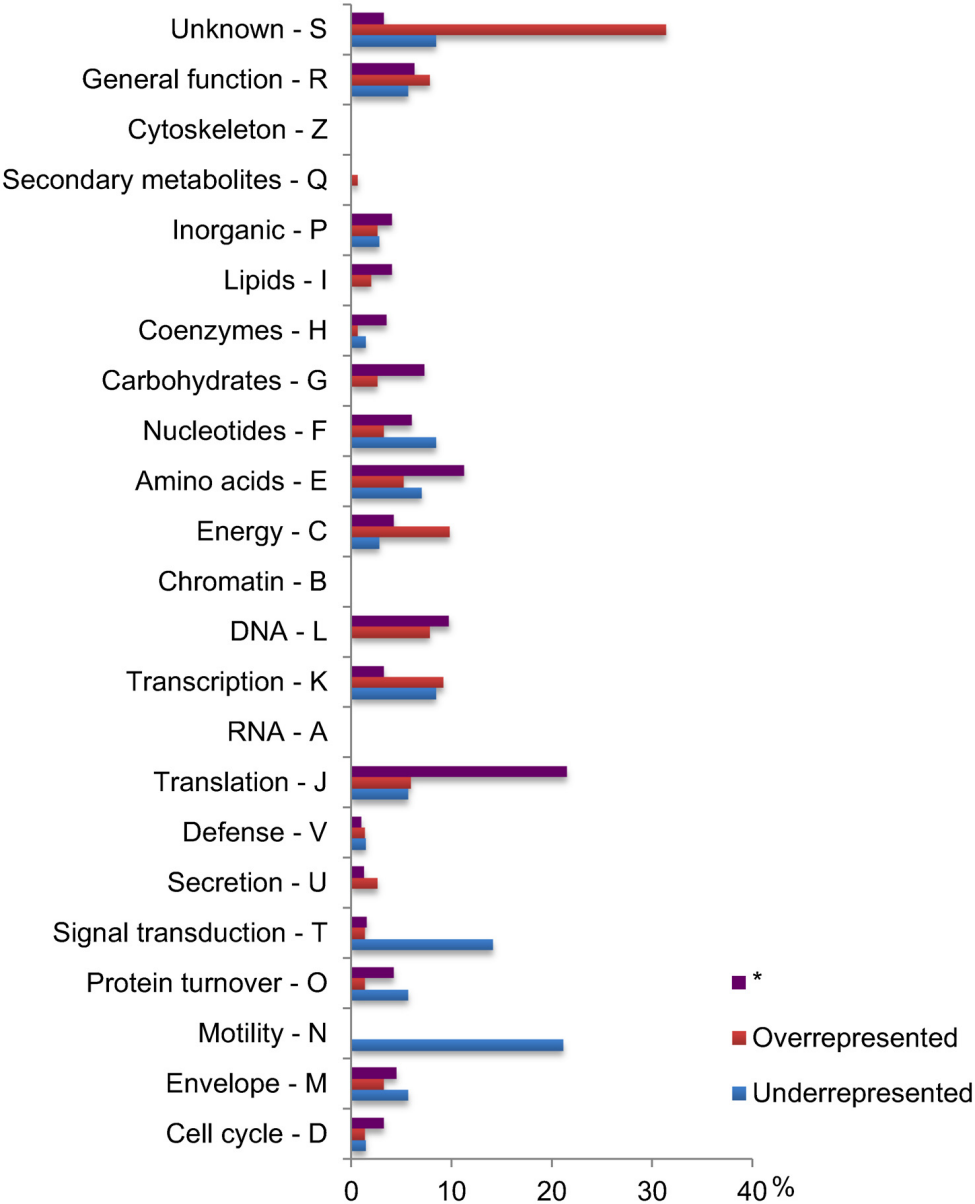
Functional composition of gut species bactNOG datasets. BactNOGs represented in one or more of the 23 gut bacterial species in this study were attributed to one of three groups: overrepresented or underrepresented compared to non-gut species (see text for details), or neither over nor underrepresented (indicated by *). Within each of these groups, the number of different BactNOGSs attributed to a functional category is indicated as a percentage of the total number of BactNOGs in the group. Functional category descriptions are short forms of the full descriptions presented in tables “S1” and “S2” Tables.

The majority of the 153 overrepresented bactNOGs have no known function, or a function involved in energy production, DNA metabolism, transcription or translation (Fig 4, S1 Table). Individual bactNOG descriptions reveal a number of functions that have earlier been associated with the gut environment, such as the degradation of conjugated bile acids (bactNOG15678, Choloylglycine hydrolase), cobalamin (vitamin B12) biosynthesis (bactNOG85989, Cobyrinic acid a, c-diamide synthase) [21] or iron acquisition (bactNOG99581, bactNOG38121, Ferric uptake regulator protein) [22, 23], emphasizing the importance of these processes in the gut environment.

Not less than 4 of the overrepresented bactNOGs point to an important role of conjugation in the gut environment (bactNOGs 07070, 26309, 08200, 44258), a function that had not been recognized as such in the earlier global approach to the identification of bacterial functions that are important in the gut environment described in [1]. Other functions that had not been recognized in the global approach are "sulfuric ester hydrolase" (sulfatase, bactNOG20561), which may play a role in the foraging of abundant sulfated glycans in the intestine, such as mucins or glycosaminoglycans [24], and "Sortase B" (bactNOG70972) which plays a role in the anchoring of a specific category of bacterial surface proteins [25].

Surprisingly, only 78 bactNOGs were shared among all the genomes in the two genome sets (Fig 3). This is far less than expected when compared with the approximately 250 bacterial protein coding genes that are considered as essential in the Gram-positive model bacterium *B*. *subtilis* [26, 27]. Also, certain bactNOGs that represent essential proteins (e.g. certain ribosomal proteins) appeared to be present in only a fraction of the genomes studied (results not shown). A closer look at the catalog of bactNOGs and their functional descriptions revealed that these observations could (in part) be explained by the fact that many functions are represented by several bactNOGs, i.e. several bactNOGs carry identical function descriptions. This is due to the way the non-supervised orthologous groups (NOGs) were constructed [18], where the authors chose for a high resolution, i.e. small precisely defined NOGs containing very similar proteins, to improve accuracy with the consequence that less similar proteins with the same function are attributed to different bactNOGs. We therefore decided to perform a second comparison of our sets of gut and non-gut genomes at the level of "groups of bactNOGs" where we considered bactNOGs with identical function descriptions as one group (in our dataset, a "group of bactNOGs" may contain from 1 to 606 bactNOGs, the latter representing "transcriptional regulator proteins"). By doing so, all the information on bactNOGs that had no description, accounting for 31.2% of the bactNOGs in our dataset, was lost. Other methods of protein clustering and protein family construction that are out of the scope of this study may be instructive in revealing the potential importance of these unclassified bactNOGs beyond the level of the single bactNOG comparisons described above [12, 28, 29].

166 functional “groups of bactNOGs” were shared among all the genomes in the two sets (data not shown). As expected, among these shared groups we find functions involved in DNA and RNA metabolism, transcription, translation, cell envelope, shape and division, as well as energy conversion and metabolism of nucleotides, coenzymes and carbohydrates.

44 groups of bactNOGs were considered as overrepresented in the genomes of the gut *Firmicutes* (Table 3). These include several functions that had already been identified in the single bactNOG comparisons described above, like "sulfuric ester hydrolase" (sulfatase, bactNOG20561), "sortase B" (bactNOG 70972), "proteins involved in unidirectional conjugation" (6 bactNOGs of which 3 had been identified in individual comparisons) and "proteins involved in conjugation with cellular fusion" (3 bactNOGs, of which 1 had been identified in individual comparisons). The latter two functions are represented in 87 and 74% of the 23 gut species, respectively, as opposed to 35 and 22% of the non-gut species (Table 3).

**Table 3:** Functional groups overrepresented in gut bacteria.

eggNOG	Function	Functional category	Abundance, %		
			egg NOG v.3.0	GUT	NONGUT
bactNOG57079	5-Aminoimidazole-4-Carboxamide ribonucleotide transformylase	[F] Nucleotide transport and metabolism	1.4	100.0	26.1
bactNOG33416	Transcriptional regulator protein-like protein	[K] Transcription	0.4	73.9	0.0
bactNOG14419, bactNOG78827	Site-Specific recombinase	NA	NA	87.0	17.4
bactNOG20561	Sulfuric ester hydrolase	[M] Cell wall/membrane/envelope biogenesis	11.1	82.6	13.0
bactNOG61174	Replication initiator protein	[S] Function unknown	0.2	69.6	0.0
bactNOG43319, bactNOG30533, bactNOG35352	Adenylate cyclase	NA	NA	78.3	13.0
bactNOG14637	Adenylosuccinate protein	[F] Nucleotide transport and metabolism	2.6	69.6	4.3
bactNOG13499	Selenate reductase subunit YgfM; with YgfK and YgfN forms a selenate reductase, which seems to catalyze the reduction of selenate to selenite; YgfM contains a FAD domain-containing protein	[C] Energy production and conversion	3.4	69.6	4.3
bactNOG78875	GB:X04470, GB:X04503, GB:X04502, SP:P03973, PID:28639, PID:338233, PID:36491, and PID:758101; identified by sequence similarity protein	[K] Transcription	0.3	65.2	0.0
bactNOG03861	Elongation factor G	[J] Translation, ribosomal structure and biogenesis	18.7	82.6	17.4
bactNOG10082	SAM dependent methyltransferase	[R] General function prediction only	7.2	82.6	17.4
bactNOG29973	Deoxycytidylate deaminase	[F] Nucleotide transport and metabolism	4.6	73.9	8.7
bactNOG20957, bactNOG28451, bactNOG18161, bactNOG37597	Specifically catalyzes the dephosphorylation of 2- phosphoglycolate. Is involved in the dissimilation of the intracellular 2-phosphoglycolate formed during the DNA repair of 3'-phosphoglycolate ends, a major class of DNA lesions induced by oxidative stress (By similarity) protein	NA	NA	95.7	34.8
bactNOG04076	Zinc phosphodiesterase, which displays some tRNA 3'- processing endonuclease activity. involved in tRNA maturation, by removing a 3'-trailer from precursor tRNA (By similarity)	[R] General function prediction only	5.1	65.2	4.3
bactNOG05123, bactNOG07417	2-Isopropylmalate synthase	NA	NA	91.3	30.4
bactNOG51505, bactNOG44758	Addiction module toxin, RelE/StbE family protein	NA	NA	73.9	13.0
bactNOG45170	Cdp-Diacylglycerol--Glycerol-3-Phosphate 3 protein	[I] Lipid transport and metabolism	5.8	73.9	13.0
bactNOG30240	Glyoxalase/Bleomycin resistance protein/Dioxygenase	[E] Amino acid transport and metabolism	2.9	69.6	8.7
bactNOG00016	Phosphoserine aminotransferase; catalyzes the formation of 3-phosphonooxypyruvate and glutamate from O-phospho-L-serine and 2-oxoglutarate; required both in major phosphorylated pathway of serine biosynthesis and in the biosynthesis of pyridoxine	[E] Amino acid transport and metabolism	41.7	95.7	39.1
bactNOG40424	Glycoside hydrolase, family 25	[M] Cell wall/membrane/envelope biogenesis	1.5	60.9	4.3
bactNOG02826	4-Alpha-Glucanotransferase	[G] Carbohydrate transport and metabolism	44.2	91.3	34.8
bactNOG03506	Aminopeptidase 2; catalyzes the removal of amino acids from the N termini of peptides	[E] Amino acid transport and metabolism	10.9	87.0	30.4
bactNOG15648, bactNOG74792	Aconitate hydratase	[C] Energy production and conversion	NA	78.3	21.7
bactNOG65104	Phosphoribosylpyrophosphate synthetase; Catalyzes the formation of PRPP from ATP and ribose 5-phosphate	[F] Nucleotide transport and metabolism	1.8	78.3	21.7
bactNOG20523	Sugar phosphatase; YidA; catalyzes the dephosphorylation of erythrose 4-phosphate (preferred substrate), mannose 1-phosphate and p-nitrophenyl phosphate; hydrolyzes the alpha-D-glucose-1-phosphate but not the beta form; member of the haloacid dehalogenase-like hydrolases superfamily and Cof family of proteins	[R] General function prediction only	10.2	78.3	21.7
bactNOG30560, bactNOG37582	Removes the formyl group from the N-terminal Met of newly synthesized proteins. Requires at least a dipeptide for an efficient rate of reaction. N-terminal L-methionine is a prerequisite for activity but the enzyme has broad specificity at other positions (By similarity)	NA	NA	69.6	13.0
bactNOG83597	Subunit C	[C] Energy production and conversion	0.4	65.2	8.7
bactNOG31052, bactNOG35249, bactNOG35454, bactNOG05302	Had-Superfamily hydrolase, subfamily IA, variant 3	NA	NA	87.0	34.8
bactNOG07070, bactNOG11507, bactNOG08200, bactNOG10025, bactNOG13178, bactNOG26309	Protein involved in unidirectional conjugation	NA	NA	87.0	34.8
bactNOG22665, bactNOG08175	Pyridoxal kinase	[H] Coenzyme transport and metabolism	NA	87.0	34.8
bactNOG74867, bactNOG16222	Sugar Hydrogen symporter protein	NA	NA	87.0	34.8
bactNOG82609	Oxaloacetate decarboxylase	[C] Energy production and conversion	21.0	78.3	26.1
bactNOG62080	Ribosomal protein S3	[J] Translation, ribosomal structure and biogenesis	0.4	60.9	8.7
bactNOG17864, bactNOG34439	RNA methyltransferase	[J] Translation, ribosomal structure and biogenesis	NA	60.9	8.7
bactNOG14801	Hydro-Lyase, Fe-S type, tartrate/fumarate subfamily, beta	[C] Energy production and conversion	8.6	91.3	39.1
bactNOG02215	Potassium transporter peripheral membrane component; involved in potassium uptake; found to be peripherally associated with the inner membrane in Escherichia coli; contains an NAD-binding domain protein	[P] Inorganic ion transport and metabolism	32.2	91.3	39.1
bactNOG45092	50S ribosomal protein L30; L30 binds domain II of the 23S rRNA and the 5S rRNA	[J] Translation, ribosomal structure and biogenesis	24.4	56.5	4.3
bactNOG70972	Sortase B	[S] Function unknown	1.7	56.5	4.3
bactNOG53104	Histidine Phosphotransfer domain-containing protein	[T] Signal transduction mechanisms	0.3	52.2	0.0
bactNOG99320	Ribosomal protein L34	[J] Translation, ribosomal structure and biogenesis	0.3	52.2	0.0
bactNOG01580	Decarboxylase, beta	[C] Energy production and conversion	15.4	82.6	30.4
bactNOG24561, bactNOG09355	L-Fucose isomerase	[G] Carbohydrate transport and metabolism	NA	73.9	21.7
bactNOG69266, bactNOG44258	Protein involved in conjugation with cellular fusion	NA	NA	73.9	21.7
bactNOG30123, bactNOG62262, bactNOG63699, bactNOG39777	Transcriptional regulator, DeoR family protein	NA	NA	73.9	21.7

bactNOGs with identical functional descriptions were grouped in our dataset. Groups of bactNOGs are presented for which the number of gut genomes where bactNOGs are represented exceeds the number of non-gut genomes where bactNOGs are represented by at least 12. Abundance, % of species where bactNOG is represented, in the eggNOG v.3.0 database (shown only for functional groups containing one bactNOG), in 23 “gut” genomes (GUT), or in 23 “non-gut” genomes (NONGUT), respectively. NA- non- assigned.

A new function which only becomes apparent with the grouped bactNOGs approach, and which again was not detected by the global approach in [1], is L-Fucose isomerase (bactNOGs 24561 and 09355), the enzyme catalizing the first step of fucose metabolism, found in 74% of the gut bacteria against 22% of the non-gut bacteria. L-Fucose is highly abundant in the intestine [30], present in dietary polysaccharides such as pectin but also in mucin glycoproteins overlying the intestinal epithelium. It can be cleaved from host glycans by multiple fucosidases produced by gut commensals such as *Bacteroides thetaiotaomicron*, resulting in a high availability in the intestinal lumen where it can be used as a carbon source by *B*. *thetaiotaomicron* itself [31] or by other resident bacteria such as *Escherichia coli* [32] or *Roseburia intestinalis* [33].

A certain number of functions that were detected as overrepresented in the single bactNOG comparisons do not appear as such in the analysis by groups of bactNOGs. Some group functions even appear in the list of overrepresented single bactNOGs (S1 Table) as well as in the list of underrepresented single bactNOGs (see below, S2 Table). This is for example the case for the function "Ferric uptake regulator protein" (represented by bactNOGs 38121 and 99581 in the list of overrepresented bactNOGs and by bactNOG31290 in the list of underrepresented bactNOGs). This type of result suggests that closely related gut and non-gut bacteria use distinct proteins for the same function or, alternatively, that the proteins represented by different bactNOGs in spite of identical descriptions exert different functions of which some are more important in the gut environment.

### Gut adaptation through functional paucity

The direct comparison of closely related bacteria from the gut and other environments also provides a unique opportunity to focus on underrepresented functions, which may be equally informative of the functioning of the gut ecosystem. In contrast to the overrepresented functions, underrepresented functions included far less bactNOGs with unknown functions (Fig 4, S2 Table). Remarkably, one third of the underrepresented bactNOGs is involved in motility and signal transduction, including several chemotaxis related functions. Another remarkable underrepresented function is the *Pur* operon repressor protein (bactNOG16918), identified in only one of the 23 gut bacteria as opposed to 21 of the 23 non-gut bacteria. This repressor controls the transcription of the pur operon for purine biosynthetic genes, and its absence would be expected to result in the constitutive transcription of the operon. The ability to synthesize nucleotides was shown to be a prerequisite for successful colonization of the mouse intestine by *E*. *coli* [34]. The constitutive expression of genes involved in purine biosynthesis may thus give a competitive advantage during unsteady nucleotide supply in the human gut environment.

The analysis of underrepresented functions by groups of bactNOGs led to the same conclusion as the single-bactNOG comparisons: of the 67 functional groups that are underrepresented in the gut bacteria (Table 4), an astonishing 1/3 appears to be involved in motility and chemotaxis. These functions are, on an average, represented in only 16% of the 23 gut bacteria studied, as opposed to 77% of the non-gut bacteria.

**Table 4:** Functional groups underrepresented in gut bacteria.

eggNOG	Function	Functional category	Abundance, %		
			egg NOG v.3.0	GUT	NONGUT
bactNOG16918	Pur operon repressor protein	[K] Transcription	15.2	4.3	91.3
bactNOG00229	Phosphoribosylaminoimidazolecarboxamide formyltransferase/IMP cyclohydrolase	[F] Nucleotide transport and metabolism	75.2	13.0	95.7
bactNOG23778, bactNOG36792, bactNOG29992, bactNOG28755, bactNOG23746, bactNOG26169, bactNOG04324, bactNOG40456, bactNOG12593	Isochorismatase	NA	NA	8.7	82.6
bactNOG01751	Flagellar biosynthesis protein FlhB; membrane protein responsible for substrate specificity switching from rod/hook-type export to filament-type export	[N] Cell motility	37.9	13.0	82.6
bactNOG00287	Involved in the modulation of the chemotaxis system; catalyzes the demethylation of specific methylglutamate residues introduced into the chemoreceptors (methyl-accepting chemotaxis proteins) by cheR	[T] Signal transduction mechanisms	39.0	13.0	82.6
bactNOG18316, bactNOG06650, bactNOG100225, bactNOG98591, bactNOG05199, bactNOG21958	Methionine sulfoxide reductase	NA	NA	13.0	82.6
bactNOG12472, bactNOG05849, bactNOG01096, bactNOG04323	Alkaline phosphatase	NA	NA	17.4	87.0
bactNOG30877, bactNOG44267	Flagellar hook capping protein	[N] Cell motility		8.7	73.9
bactNOG00716, bactNOG85469, bactNOG30379	Flagellin protein	NA	NA	17.4	82.6
bactNOG43795	Protein FliQ	[N] Cell motility	30.4	17.4	82.6
bactNOG20038	Cell envelope-related transcriptional attenuator; TIGRFAM: cell envelope-related function transcriptional attenuator, LytR/CpsA family; PFAM: cell envelope-related transcriptional attenuator protein	[K] Transcription	12.7	21.7	87.0
bactNOG28010, bactNOG02636	Ribose-Phosphate pyrophosphokinase	NA	NA	30.4	95.7
bactNOG52752, bactNOG42812, bactNOG27823, bactNOG44224 bactNOG26630	Chec, inhibitor of MCP methylation protein	NA	NA	21.7	82.6
bactNOG30371, bactNOG95849, bactNOG30633	Flagellar basal body rod protein	[N] Cell motility		21.7	82.6
bactNOG04544	Flagellar basal body rod protein FlgG	[N] Cell motility	41.4	21.7	82.6
bactNOG02669	Flagellar biosynthesis protein FlhA	[N] Cell motility	43.8	21.7	82.6
bactNOG02127	Flagellar biosynthesis protein FliP; FliP, with proteins FliQ and FliR, forms the core of the central channel in the flagella export apparatus	[N] Cell motility	42.3	21.7	82.6
bactNOG97172, bactNOG100574, bactNOG19021, bactNOG22405, bactNOG34382, bactNOG38432, bactNOG98529, bactNOG75984, bactNOG10394, bactNOG03185	Flagellar hook-associated protein	NA	NA	21.7	82.6
bactNOG50628, bactNOG37469, bactNOG36391, bactNOG46261	Flagellar hook-basal body protein	NA	NA	21.7	82.6
bactNOG10389, bactNOG44208, bactNOG02345	Flagellar motor switch protein	NA	NA	21.7	82.6
bactNOG38595, bactNOG34666, bactNOG43852	Flagellar protein FliS	NA	NA	21.7	82.6
bactNOG02069	Flagellum-Specific ATP synthase	[C] Energy production and conversion	43.2	21.7	82.6
bactNOG37514, bactNOG31243, bactNOG42066, bactNOG27248, bactNOG09558, bactNOG14057, bactNOG72426	Protein involved in cellular iron ion homeostasis	NA	NA	21.7	82.6
bactNOG27648, bactNOG36097, bactNOG01870, bactNOG31082, bactNOG74955, bactNOG16925, bactNOG31591, bactNOG06736, bactNOG00914, bactNOG25916, bactNOG01033, bactNOG15932, bactNOG37025, bactNOG40000, bactNOG32982, bactNOG20199	Protein involved in chemotaxis	NA	NA	21.7	82.6
bactNOG43731	50S ribosomal protein L34; in Escherichia coli transcription of this gene is enhanced by polyamines	[J] Translation, ribosomal structure and biogenesis	63.9	34.8	95.7
bactNOG00965	Phosphoribosylaminoimidazole carboxylase ATPase subunit	[F] Nucleotide transport and metabolism	53.4	0.0	60.9
bactNOG05379, bactNOG02517, bactNOG19673, bactNOG01018	Udp-N-Acetylglucosamine 2-epimerase	NA	NA	34.8	95.7
bactNOG16773, bactNOG00811	Atp-Dependent protease	NA	NA	4.3	65.2
bactNOG00957	Catalyzes the condensation of the acetyl group of acetyl-CoA with 3-methyl-2-oxobutanoate (2-oxoisovalerate) to form 3-carboxy-3-hydroxy-4-methylpentanoate protein	[E] Amino acid transport and metabolism	49.1	4.3	65.2
bactNOG25456	Glutamine amidotransferase, subunit PdxT; with PdxST is involved in the biosynthesis of pyridoxal 5'-phosphate; PdxT catalyzes the hydrolysis of glutamine to glutamate and ammonia; PdxS utilizes the ammonia to synthesize pyridoxal 5'-phosphate	[H] Coenzyme transport and metabolism	17.4	4.3	65.2
bactNOG13897, bactNOG00750, bactNOG67445, bactNOG14611, bactNOG60045, bactNOG74366, bactNOG15012, bactNOG65815, bactNOG37933, bactNOG59733, bactNOG19072, bactNOG20172, bactNOG28001	Methyl-Accepting chemotaxis sensory transducer protein	NA	NA	4.3	65.2
bactNOG12090	Methyltransferase, CheR	[N] Cell motility/ [T] Signal transduction mechanisms	5.0	4.3	65.2
bactNOG52841, bactNOG46445, bactNOG96629, bactNOG90266	Flagellar export protein FliJ	NA	NA	8.7	69.6
bactNOG48374, bactNOG99922, bactNOG82249, bactNOG13602, bactNOG80514	Protein involved in flagellum assembly	NA	NA	8.7	69.6
bactNOG01470, bactNOG04846	Cell division protein FtsA	NA	NA	26.1	82.6
bactNOG02318	Flagellar motor switch protein FliM	[N] Cell motility	41.9	21.7	78.3
bactNOG05792	Cyanophycin synthetase	[M] Cell wall/membrane/envelope biogenesis	18.5	0.0	56.5
bactNOG29500, bactNOG10264, bactNOG18792	Protein involved in cytochrome complex assembly	NA	NA	0.0	56.5
bactNOG92570, bactNOG97494, bactNOG46066	Ribosomal protein L30	NA	NA	34.8	91.3
bactNOG11597, bactNOG13922	Ribonuclease Z; member of metallo-beta-lactamase family; the purified enzyme from Escherichia coli forms dimeric zinc phosphodiesterase; in Bacillus subtilis this protein is a 3'-tRNA processing endoribonuclease and is essential while in Escherichia coli it is not; associates with two zinc ions	NA	NA	4.3	60.9
bactNOG37372	Stage II sporulation protein M	[S] Function unknown	6.6	4.3	60.9
bactNOG46911	Transcriptional regulator, CopG family protein	[K] Transcription	6.3	4.3	60.9
bactNOG42002, bactNOG86985, bactNOG92617, bactNOG50625, bactNOG75642, bactNOG89894, bactNOG92834, bactNOG48059	Type IV pilus assembly PilZ protein	NA	NA	8.7	65.2
bactNOG31141, bactNOG26612, bactNOG36412, bactNOG37092, bactNOG71934, bactNOG52195	CBS domain-containing protein	NA	NA	13.0	69.6
bactNOG01343	Plays an important role in the de novo pathway of purine nucleotide biosynthesis protein	[F] Nucleotide transport and metabolism	76.7	30.4	87.0
bactNOG12565	Atp:Guanido phosphotransferase	[E] Amino acid transport and metabolism	12.7	21.7	73.9
bactNOG44690, bactNOG74103, bactNOG55341, bactNOG53379	Carbon storage regulator protein	NA	NA	26.1	78.3
bactNOG00578	Drug resistance transporter, EmrB/QacA protein	[P] Inorganic ion transport and metabolism	56.8	26.1	78.3
bactNOG67310, bactNOG67983, bactNOG64693, bactNOG67454, bactNOG33438	Enzyme activator	NA	NA	21.7	73.9
bactNOG01697, bactNOG24497, bactNOG05093	Sigma factors are initiation factors that promote the attachment of RNA polymerase to specific initiation sites and are then released	NA	NA	26.1	78.3
bactNOG28544, bactNOG33086	3-Methyladenine DNA glycosylase	NA	NA	0.0	52.2
bactNOG35415, bactNOG66375, bactNOG32336, bactNOG28346	Glycerol-3-Phosphate responsive antiterminator protein	NA	NA	0.0	52.2
bactNOG19498, bactNOG43068, bactNOG11337	Phenylalanine-Trna ligase	NA	NA	0.0	52.2
bactNOG04772, bactNOG01433, bactNOG09984	Peptidase M16	NA	NA	39.1	91.3
bactNOG16417	Flagellar motor protein MotD; Homologous to MotB. These organism have both MotB and MotD. With MotC (a MotA homolog) forms the ion channels that couple flagellar rotation to proton/sodium motive force across the membrane and forms the stator elements of the rotary flagellar machine. Either MotAB or MotCD is sufficient for swimming, but both are necessary for swarming motility	[N] Cell motility	13.8	4.3	56.5
bactNOG55225, bactNOG27515, bactNOG65818, bactNOG01779, bactNOG10628	Iron-Sulfur cluster-binding protein	NA	NA	4.3	56.5
bactNOG87479, bactNOG49851 bactNOG55890, bactNOG86277 bactNOG102115, bactNOG33628 bactNOG16540, bactNOG53787	TPR repeat-containing protein	NA	NA	4.3	56.5
bactNOG00626	Arsenical-Resistance protein	[P] Inorganic ion transport and metabolism	29.8	8.7	60.9
bactNOG50959, bactNOG48759, bactNOG89587, bactNOG39189, bactNOG43920, bactNOG36618, bactNOG38281, bactNOG52031, bactNOG51626, bactNOG95758	Glutaredoxin protein	NA	NA	8.7	60.9
bactNOG00525	Utp-Glucose-1-Phosphate uridylyltransferase	[M] Cell wall/membrane/envelope biogenesis	63.7	30.4	82.6
bactNOG18657	Bifunctional pyrimidine regulatory protein PyrR uracil phosphoribosyltransferase; regulates pyrimidine biosynthesis by binding to the mRNA of the pyr genes, also has been shown to have uracil phosphoribosyltransferase activity	[F] Nucleotide transport and metabolism	35.0	17.4	69.6
bactNOG01465	Flagellar hook protein FlgE	[N] Cell motility	41.7	13.0	65.2
bactNOG04205, bactNOG34242	Ppx/Gppa phosphatase	NA	NA	13.0	65.2
bactNOG44112, bactNOG36504	RNA chaperone that binds small regulatory RNA (sRNAs) and mRNAs to facilitate mRNA translational regulation in response to envelope stress, environmental stress and changes in metabolite concentrations. Also binds with high specificity to tRNAs protein	NA	NA	17.4	69.6
bactNOG01716	Undecaprenyl-Phosphate alpha-N protein	[M] Cell wall/membrane/envelope biogenesis	41.4	17.4	69.6
bactNOG14292, bactNOG05550, bactNOG03912, bactNOG60601, bactNOG03248, bactNOG63252, bactNOG10183, bactNOG00751, bactNOG00172, bactNOG58297	Atp-Dependent helicase	NA	NA	34.8	87.0
bactNOG23776, bactNOG28510 bactNOG86058, bactNOG60688 bactNOG38850, bactNOG11515, bactNOG02006, bactNOG58574, bactNOG85199, bactNOG08921, bactNOG35523, bactNOG34604, bactNOG10155, bactNOG18901, bactNOG09956, bactNOG74830, bactNOG34667, bactNOG21541, bactNOG32868, bactNOG02188, bactNOG27650, bactNOG43067, bactNOG33646, bactNOG30150, bactNOG09220, bactNOG74004, bactNOG87394, bactNOG08118, bactNOG87029, bactNOG29496, bactNOG50314, bactNOG42983, bactNOG42538, bactNOG24960, bactNOG12392, bactNOG09967, bactNOG05219, bactNOG10337, bactNOG02508	Diguanylate cyclase	NA	NA	34.8	87.0

bactNOGs with identical functional descriptions were grouped in our dataset. Groups of bactNOGs are presented for which the number of non-gut genomes where bactNOGs are represented exceeds the number of gut genomes where bactNOGs are represented by at least 12. Abundance, % of species where bactNOG is represented, in the eggNOG v.3.0 database (shown only for functional groups containing one bactNOG), in 23 gut genomes (GUT), or in 23 non-gut genomes (NONGUT), respectively. NA- non- assigned.

### Underrepresentation of secreted proteins in gut bacteria

**Fig 5:**
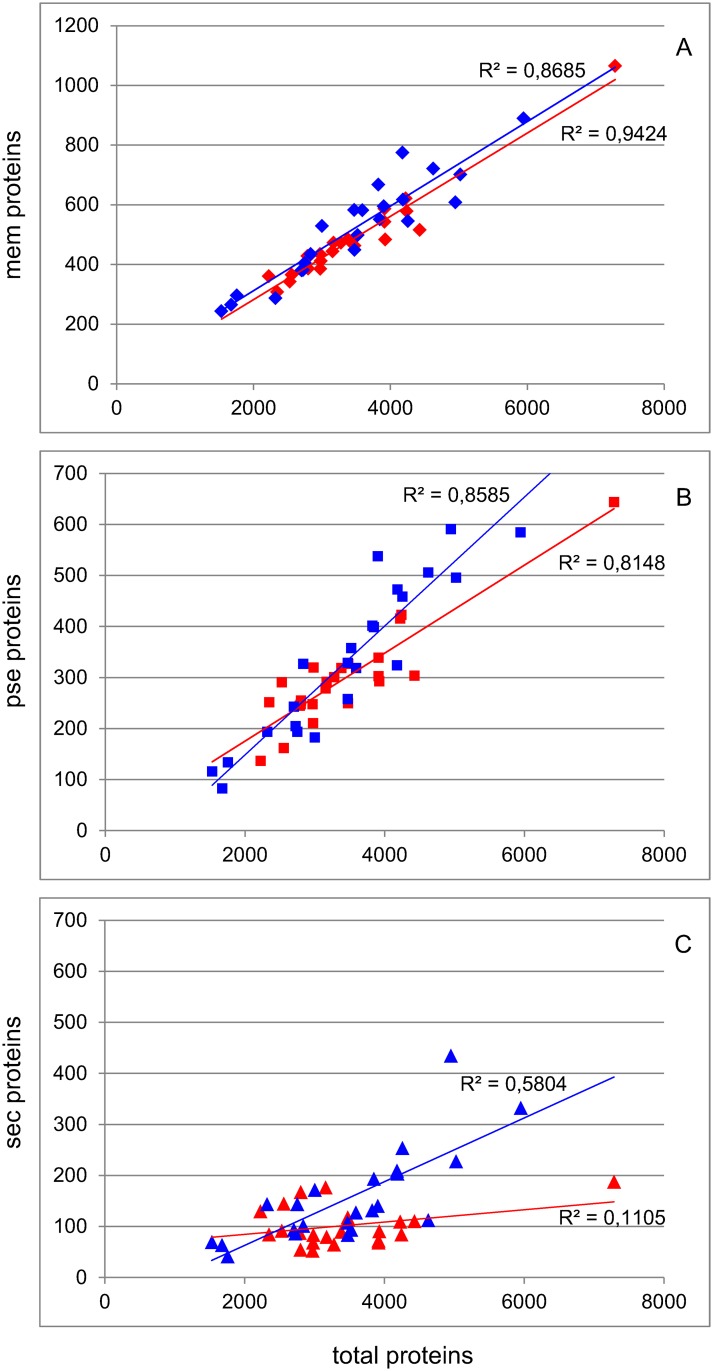
Predicted numbers of bacterial membrane, potentially surface exposed and secreted proteins as a function of the total number of proteins. The predicted numbers of (A) membrane (mem), (B) potentially surface exposed (pse), and (C) secreted (sec) proteins in a bacterial species are correlated to the total number of encoded proteins (Spearman's rank correlation test, p < 0.01), with the exception of the sec proteins in gut bacteria where no significant correlation is observed (p > 0.2). Red, gut bacteria; blue, non-gut bacteria.

The predicted proteomes of the strains in this study were analyzed using SurfG+ [19] to predict protein localization (Table 2). This analysis revealed a difference in the numbers of secreted proteins where on an average 3.1% (SEM 0.3) of proteins were predicted to be secreted in our set of gut bacteria as opposed to 4.3% (SEM 0.3) for the non-gut bacteria (not shown). Interestingly, this difference appears to be explained by the presence of relatively low and stable numbers of secreted proteins across differently sized gut bacterial genomes, while in the closely related non-gut bacteria the number of secreted proteins clearly correlates with the total number of proteins encoded in the genome (Fig 5). The numbers of predicted membrane proteins and surface exposed proteins are correlated to the total numbers of encoded proteins in both gut and non-gut bacteria (Fig 5).

## Discussion

The human gut microbiota is increasingly recognized as a major health determining factor. As our knowledge on this microbial community and notably its bacterial component expands, it becomes clear that atypical microbiota compositions, dysbioses, are associated with a growing number of diseases, to an extent that microbiota composition can constitute a "signature" or bio-marker of a specific disease (e.g. [35, 36]). At least for some diseases, experiments in animals convincingly show that an atypical microbiota can be a driving force in the development of disease. In line with these observations, promising results have been reported with the use of fecal microbiota transplantation (in this case the transfer of fecal material from a healthy donor to the intestine of a patient) to improve the symptoms of inflammatory bowel disease in humans [37].

Yet, our knowledge on the bacterial properties that drive homeostasis, the equilibrium of the gut microbial ecosystem including bacteria—host interactions, is still limited. Seen from the bacterial side, which are the genes a bacterium needs to maintain itself in this ecosystem? Which are the bacterial genes that play a role in the functioning of the system, including interactions with the host, as a whole? Several approaches, *in silico* and through experimental screening, have been and are used to answer these questions. One of the main *in silico* approaches consists of a global comparison of functions encoded by the gut bacteria and functions encoded by non-gut bacteria, looking for what seems to be gut-specific, as examplified by the study presented in [1]. A possible drawback of this approach, however, is that the taxonomical composition of the gut microbiota and the non-gut reference data set may be largely different. As a consequence, observed differences may in part be due to inherent differences between bacterial taxons that are not necessarily relevant for the comprehension of the gut ecosystem. On the other hand, this approach may fail to detect obvious gut adaptations in a specific taxon if similar functions exist in a different taxon among the non-gut bacteria.

In the present study we therefore used a complementary approach and compared data from selected gut bacteria and closely related non-gut bacteria of the *Firmicutes* phylum, a procedure that should favor the detection of environment-specific adaptations. We observed a tendency of a relatively low and stable number of predicted secreted proteins across the gut bacteria that was not observed in the non-gut bacteria. It will be interesting to see if this tendency is confirmed when larger numbers of genomes will be analyzed. If so, this could mean that gut bacteria limit the number of secreted proteins, maybe in response to the intestinal flow as this type of proteins could easily become separated from, and thus be of limited advantage to, the secreting bacteria.

We identified several functions that may play an important role in the gut environment but had gone undetected by the global comparison approach described in [1]. For instance, our data strongly suggest that conjugation plays an important role in the gut environment. Conjugation is the most effective mechanism of horizontal gene transfer (HGT) where the exchange of genetic material can occur even between highly divergent bacterial species [38], and our conclusion is in line with earlier evidence of HGT in the gut environment [39–41]. The ability to acquire fitness genes by conjugation may provide gut bacteria with competitive advantages to thrive in this complex environment. Of clinical importance, elevated bacterial conjugation activity in the densely populated gut ecosystem, an environment recognized as a significant reservoir of antibiotic resistances [42], may also play an important role in the spread of antibiotic resistance genes. We further detected sulfatase and L-fucose isomerase as overrepresented functions in the gut bacteria. Sulfatases and their role in the foraging of abundant sulfated glycans in the gut have been described as critical for the fitness of *Bacteroides thetaiotaomicron* in the gut environment [24], but are far less studied in Firmicutes [24, 43]. Similarly, L-fucose isomerase is involved in the metabolism of L-fucose, a highly abundant sugar in the intestine [30], present in dietary polysaccharides such as pectin but also in mucin glycoproteins overlying the intestinal epithelium.

Together, these examples clearly illustrate the potential of our targeted comparative analysis, focusing on closely related bacteria from different environments, to identify functions that are important in the gut environment. This approach also permits to identify functions that are underrepresented among gut bacteria, an analysis that proved equally informative. We thus observed that an astonishing one third of the underrepresented functions appears to be involved in motility and chemotaxis, representing to our knowledge the first observation of this kind. Bacterial chemotaxis is the phenomenon whereby bacteria direct their movements according to certain chemical stimulants in their environment, and our observation may be explained by the fact that the majority of the bacteria from the “non-gut” set were isolated from water or soil. It is easy to imagine that in these environments it is important for bacteria to move towards the highest concentration of food or other essential molecules, or to flee from poisons. In the gut, the opposite is true as free molecules in transit pass by bacteria that are often adhering to the intestinal surfaces or food particles [44]. An alternative explication may be that these commensal bacteria have been selected for the absence of one of the best known immune modulatory bacterial cell surface proteins, flagellin, that interacts with TLR5 to induce an inflammatory response [45].

The present study can be regarded as a proof of principle demonstrating the potential of taxonomically targeted comparative analyses in the identification of functions that are important in a given ecosystem, in our case the human intestinal tract. The results of these analyses confirmed a number of earlier observations or intuitions about functions that are considered as key functions in the gut environment. The analyses not only identified new functions but also a relative paucity in some other functions, both of which appear to be important in the human gut environment and that, even if experimental evidence is still incomplete, intuitively seem to make sense. These results suggest that the identified "unknown functions" that are found to be overrepresented in the gut bacteria in our analysis are important too and worth further investigating.

In this pilot experiment we limited ourselves to completely sequenced genomes. Without this self-imposed limitation, which is probably not necessary, a wealth of additional data becomes available for analysis. Many more bacterial genomes have been sequenced to near completion since we started this study and ever more are becoming available, including "metagenomic species" genomes that are directly assembled from metagenomic data [46]. The use of these data will allow more robust studies with higher numbers of bacteria. Parallel developments see the pairwise comparison of two human gut microbiota types, typically patients and healthy subjects, rather than comparison of the gut microbiota with bacteria from completely different ecosystems. While not answering exactly the same questions, the different approaches are complementary and should together eventually lead to the unraveling of the critical factors in gut homeostasis that rule our health. The acquired knowledge may subsequently guide our choice of health-beneficial probiotics, screening for desired properties to restore or consolidate homeostasis and avoiding properties that are incompatible with homeostasis.

## Supporting Information

S1 Table BactNOGs overrepresented in gut bacteria. (DOCX)

S2 Table BactNOGs underrepresented in gut bacteria. (DOCX)
