# Supplementary material for: Functional Comparison of Bacteria from the Human Gut and Closely Related Non-Gut Bacteria Reveals the Importance of Conjugation and a Paucity of Motility and Chemotaxis Functions in the Gut Environment
Source: PLoS One. 2016 Jul 14;11(7):e0159030. doi: 10.1371/journal.pone.0159030 (PMC4945068; doi:10.1371/journal.pone.0159030)
Supplement: S1 Table — (DOCX) [file pone.0159030.s001.docx]

**S1 Table. BactNOGs overrepresented in gut bacteria**

| **eggNOG** | **Function** | **Functional category** | **Abundance, %** | | |
| --- | --- | --- | --- | --- | --- |
|  |  |  | **egg NOG v.3.0** | **GUT** | **NONGUT** |
| bactNOG01842 | Required for the thiolation of cytidine in position 32 of tRNA, to form 2-thiocytidine (s(2)C32) protein | [D] Cell cycle control, cell division, chromosome partitioning | 41.6 | 91.3 | 39.1 |
| bactNOG85989 | Cobyrinic acid ac-diamide synthase | [D] Cell cycle control, cell division, chromosome partitioning | 0.3 | 78.3 | 13.0 |
| bactNOG20561 | Sulfuric ester hydrolase | [M] Cell wall/membrane/envelope biogenesis | 11.1 | 82.6 | 13.0 |
| bactNOG03140 | Mechanosensitive ion channel | [M] Cell wall/membrane/envelope biogenesis | 33.1 | 69.6 | 8.7 |
| bactNOG15678 | Choloylglycine hydrolase | [M] Cell wall/membrane/envelope biogenesis | 4.6 | 65.2 | 8.7 |
| bactNOG40424 | Glycoside hydrolase, family 25 | [M] Cell wall/membrane/envelope biogenesis | 1.5 | 60.9 | 4.3 |
| bactNOG48259 |  | [M] Cell wall/membrane/envelope biogenesis | 0.4 | 60.9 | 4.3 |
| bactNOG42796 | Heat shock protein | [O] Posttranslational modification, protein turnover, chaperones | 1.9 | 60.9 | 0.0 |
| bactNOG99093 |  | [O] Posttranslational modification, protein turnover, chaperones/[U] Intracellular trafficking, secretion, and vesicular transport | 2.0 | 73.9 | 8.7 |
| bactNOG85818 | Manganese ion binding protein | [T] Signal transduction mechanisms | 0.2 | 56.5 | 4.3 |
| bactNOG53104 | Histidine Phosphotransfer domain-containing protein | [T] Signal transduction mechanisms | 0.3 | 52.2 | 0.0 |
| bactNOG07070 | Protein involved in unidirectional conjugation | [U] Intracellular trafficking, secretion, and vesicular transport | 14.7 | 87.0 | 30.4 |
| bactNOG49674 |  | [U] Intracellular trafficking, secretion, and vesicular transport | 1.6 | 82.6 | 26.1 |
| bactNOG15997 |  | [U] Intracellular trafficking, secretion, and vesicular transport | 1.2 | 65.2 | 4.3 |
| bactNOG85267 |  | [U] Intracellular trafficking, secretion, and vesicular transport | 0.3 | 65.2 | 8.7 |
| bactNOG08694 | Protein involved in drug transmembrane transport | [V] Defense mechanisms | 1.8 | 69.6 | 4.3 |
| bactNOG01198 | Abc-Transporter protein | [V] Defense mechanisms | 2.4 | 52.2 | 0.0 |
| bactNOG02252 | Gtpase | [J] Translation, ribosomal structure and biogenesis | 4.1 | 87.0 | 34.8 |
| bactNOG03861 | Elongation factor G | [J] Translation, ribosomal structure and biogenesis | 18.7 | 82.6 | 17.4 |
| bactNOG03322 | Catalyzes the attachment of glutamate to tRNA in a two-step reaction: glutamate is first activated by ATP to form Glu-AMP and then transferred to the acceptor end of tRNA protein | [J] Translation, ribosomal structure and biogenesis | 4.1 | 73.9 | 21.7 |
| bactNOG37582 | Removes the formyl group from the N-terminal Met of newly synthesized proteins. Requires at least a dipeptide for an efficient rate of reaction. N-terminal L-methionine is a prerequisite for activity but the enzyme has broad specificity at other positions (By similarity) | [J] Translation, ribosomal structure and biogenesis | 10.3 | 65.2 | 4.3 |
| bactNOG18524 | One of the primary rRNA binding proteins, this protein initially binds near the 5'-end of the 23S rRNA. It is important during the early stages of 50S assembly. It makes multiple contacts with different domains of the 23S rRNA in the assembled 50S subunit and ribosome (By similarity) | [J] Translation, ribosomal structure and biogenesis | 10.6 | 60.9 | 8.7 |
| bactNOG62080 | Ribosomal protein S3 | [J] Translation, ribosomal structure and biogenesis | 0.4 | 60.9 | 8.7 |
| bactNOG76728 |  | [J] Translation, ribosomal structure and biogenesis | 0.5 | 60.9 | 8.7 |
| bactNOG45092 | 50S ribosomal protein L30; L30 binds domain II of the 23S rRNA and the 5S rRNA | [J] Translation, ribosomal structure and biogenesis | 24.4 | 56.5 | 4.3 |
| bactNOG99320 | Ribosomal protein L34 | [J] Translation, ribosomal structure and biogenesis | 0.3 | 52.2 | 0.0 |
| bactNOG46051 |  | [K] Transcription | 3.3 | 82.6 | 17.4 |
| bactNOG70218 |  | [K] Transcription | 0.4 | 82.6 | 4.3 |
| bactNOG41929 | Cyclic nucleotide-binding protein | [K] Transcription | 2.5 | 78.3 | 26.1 |
| bactNOG62729 | Influences transcription termination and antitermination. Acts as a component of the transcription complex, and interacts with the termination factor rho and RNA polymerase (By similarity) | [K] Transcription | 0.5 | 78.3 | 26.1 |
| bactNOG33416 | Transcriptional regulator protein-like protein | [K] Transcription | 0.4 | 73.9 | 0.0 |
| bactNOG38121 | Ferric uptake regulator protein | [K] Transcription | 4.8 | 73.9 | 13.0 |
| bactNOG08834 | Helix-Turn-Helix- domain containing protein, AraC type | [K] Transcription | 3.4 | 65.2 | 13.0 |
| bactNOG30123 | Transcriptional regulator, DeoR family protein | [K] Transcription | 3.3 | 65.2 | 13.0 |
| bactNOG50188 |  | [K] Transcription | 0.5 | 65.2 | 8.7 |
| bactNOG78875 | GB:X04470, GB:X04503, GB:X04502, SP:P03973, PID:28639, PID:338233, PID:36491, and PID:758101; identified by sequence similarity protein | [K] Transcription | 0.3 | 65.2 | 0.0 |
| bactNOG99581 | Ferric uptake regulator protein | [K] Transcription | 0.4 | 65.2 | 8.7 |
| bactNOG23019 | Transcriptional regulator, AraC family protein | [K] Transcription | 2.5 | 60.9 | 4.3 |
| bactNOG65452 | Transcriptional regulator protein | [K] Transcription | 0.4 | 60.9 | 0.0 |
| bactNOG41236 | Transcriptional regulator, GntR family protein | [K] Transcription | 2.0 | 52.2 | 0.0 |
| bactNOG14419 | Site-Specific recombinase | [L] Replication, recombination and repair | 1.9 | 87.0 | 17.4 |
| bactNOG12111 | Recombinase | [L] Replication, recombination and repair | 3.0 | 82.6 | 17.4 |
| bactNOG14604 |  | [L] Replication, recombination and repair | 2.3 | 78.3 | 13.0 |
| bactNOG01841 | Protein involved in methylation | [L] Replication, recombination and repair | 6.6 | 73.9 | 13.0 |
| bactNOG22094 |  | [L] Replication, recombination and repair | 6.0 | 73.9 | 21.7 |
| bactNOG26309 | Protein involved in unidirectional conjugation | [L] Replication, recombination and repair | 2.6 | 73.9 | 0.0 |
| bactNOG62938 | Helix-Turn-Helix XRE-family like proteins | [L] Replication, recombination and repair | 0.2 | 73.9 | 4.3 |
| bactNOG02504 | Damaged DNA binding protein | [L] Replication, recombination and repair | 4.9 | 69.6 | 8.7 |
| bactNOG31948 | Mutt/Nudix family protein | [L] Replication, recombination and repair | 1.1 | 69.6 | 4.3 |
| bactNOG64478 | Integrase | [L] Replication, recombination and repair | 0.3 | 69.6 | 0.0 |
| bactNOG08200 | Protein involved in unidirectional conjugation | [L] Replication, recombination and repair | 7.2 | 60.9 | 8.7 |
| bactNOG90483 | Integrase | [L] Replication, recombination and repair | 0.2 | 60.9 | 0.0 |
| bactNOG08525 | Rubrerythrin protein | [C] Energy production and conversion | 3.6 | 95.7 | 43.5 |
| bactNOG08622 | Protein involved in iron-sulfur cluster assembly | [C] Energy production and conversion | 2.5 | 95.7 | 30.4 |
| bactNOG14801 | Hydro-Lyase, Fe-S type, tartrate/fumarate subfamily, beta | [C] Energy production and conversion | 8.6 | 91.3 | 39.1 |
| bactNOG01580 | Decarboxylase, beta | [C] Energy production and conversion | 15.4 | 82.6 | 30.4 |
| bactNOG05544 | Na+/H+ antiporter protein | [C] Energy production and conversion | 11.9 | 78.3 | 17.4 |
| bactNOG15221 | Hydrogenase | [C] Energy production and conversion | 3.7 | 78.3 | 21.7 |
| bactNOG82609 | Oxaloacetate decarboxylase | [C] Energy production and conversion | 21.0 | 78.3 | 26.1 |
| bactNOG15648 | Aconitate hydratase | [C] Energy production and conversion | 3.9 | 73.9 | 21.7 |
| bactNOG04083 | Isocitrate dehydrogenase | [C] Energy production and conversion | 14.8 | 69.6 | 13.0 |
| bactNOG13499 | Selenate reductase subunit YgfM; with YgfK and YgfN forms a selenate reductase, which seems to catalyze the reduction of selenate to selenite; YgfM contains a FAD domain-containing protein | [C] Energy production and conversion | 3.4 | 69.6 | 4.3 |
| bactNOG31533 | 2Fe-2S-Binding domain protein | [C] Energy production and conversion | 1.1 | 69.6 | 8.7 |
| bactNOG32286 | Flavodoxin protein | [C] Energy production and conversion | 1.8 | 65.2 | 4.3 |
| bactNOG83597 | Subunit C | [C] Energy production and conversion | 0.4 | 65.2 | 8.7 |
| bactNOG21403 | Anaerobic ribonucleoside-triphosphate reductase activating protein | [C] Energy production and conversion | 4.0 | 60.9 | 4.3 |
| bactNOG57617 | Produces ATP from ADP in the presence of a proton gradient across the membrane. The gamma chain is believed to be important in regulating ATPase activity and the flow of protons through the CF(0) complex | [C] Energy production and conversion | 0.2 | 60.9 | 4.3 |
| bactNOG00016 | Phosphoserine aminotransferase; catalyzes the formation of 3-phosphonooxypyruvate and glutamate from O-phospho-L-serine and 2-oxoglutarate; required both in major phosphorylated pathway of serine biosynthesis and in the biosynthesis of pyridoxine | [E] Amino acid transport and metabolism | 41.7 | 95.7 | 39.1 |
| bactNOG03506 | Aminopeptidase 2; catalyzes the removal of amino acids from the N termini of peptides | [E] Amino acid transport and metabolism | 10.9 | 87.0 | 30.4 |
| bactNOG05123 | 2-Isopropylmalate synthase | [E] Amino acid transport and metabolism | 22.5 | 87.0 | 26.1 |
| bactNOG98018 | Aspartate-Semialdehyde dehydrogenase | [E] Amino acid transport and metabolism | 4.8 | 82.6 | 21.7 |
| bactNOG07187 | Transcriptional regulator, GntR family protein | [E] Amino acid transport and metabolism | 2.7 | 78.3 | 13.0 |
| bactNOG01172 | Peptidase M24 | [E] Amino acid transport and metabolism | 24.9 | 73.9 | 13.0 |
| bactNOG30240 | Glyoxalase/Bleomycin resistance protein/Dioxygenase | [E] Amino acid transport and metabolism | 2.9 | 69.6 | 8.7 |
| bactNOG49567 | Oxidoreductase, acting on single donors with incorporation of molecular oxygen, incorporation of two atoms of oxygen | [E] Amino acid transport and metabolism | 0.6 | 60.9 | 8.7 |
| bactNOG57079 | 5-Aminoimidazole-4-Carboxamide ribonucleotide transformylase | [F] Nucleotide transport and metabolism | 1.4 | 100.0 | 26.1 |
| bactNOG65104 | Phosphoribosylpyrophosphate synthetase; Catalyzes the formation of PRPP from ATP and ribose 5-phosphate | [F] Nucleotide transport and metabolism | 1.8 | 78.3 | 21.7 |
| bactNOG29973 | Deoxycytidylate deaminase | [F] Nucleotide transport and metabolism | 4.6 | 73.9 | 8.7 |
| bactNOG14637 | Adenylosuccinate protein | [F] Nucleotide transport and metabolism | 2.6 | 69.6 | 4.3 |
| bactNOG98544 | Catalyzes the transfer of a ribosyl phosphate group from 5-phosphoribose 1-diphosphate to orotate, leading to the formation of orotidine monophosphate (OMP) protein | [F] Nucleotide transport and metabolism | 1.1 | 56.5 | 4.3 |
| bactNOG02826 | 4-Alpha-Glucanotransferase | [G] Carbohydrate transport and metabolism | 44.2 | 91.3 | 34.8 |
| bactNOG05382 | Sucrose-6-Phosphate hydrolase | [G] Carbohydrate transport and metabolism | 24.8 | 78.3 | 26.1 |
| bactNOG16236 | 1-Phosphofructokinase | [G] Carbohydrate transport and metabolism | 3.9 | 69.6 | 17.4 |
| bactNOG12437 | Part of a binding-protein-dependent transport system. responsible for the translocation of the substrate across the membrane (By similarity) | [G] Carbohydrate transport and metabolism | 1.3 | 60.9 | 8.7 |
| bactNOG08175 | Pyridoxal kinase | [H] Coenzyme transport and metabolism | 6.6 | 73.9 | 21.7 |
| bactNOG45170 | Cdp-Diacylglycerol--Glycerol-3-Phosphate 3 protein | [I] Lipid transport and metabolism | 5.8 | 73.9 | 13.0 |
| bactNOG45866 | Biotin/Lipoyl attachment domain-containing protein | [I] Lipid transport and metabolism | 1.5 | 69.6 | 4.3 |
| bactNOG01322 | Phospholipase D | [I] Lipid transport and metabolism | 18.1 | 60.9 | 4.3 |
| bactNOG02215 | Potassium transporter peripheral membrane component; involved in potassium uptake; found to be peripherally associated with the inner membrane in Escherichia coli; contains an NAD-binding domain protein | [P] Inorganic ion transport and metabolism | 32.2 | 91.3 | 39.1 |
| bactNOG01039 | Potassium uptake protein | [P] Inorganic ion transport and metabolism | 37.2 | 87.0 | 30.4 |
| bactNOG76941 | Heavy metal transport/detoxification protein | [P] Inorganic ion transport and metabolism | 1.0 | 65.2 | 4.3 |
| bactNOG15267 | Phosphate protein | [P] Inorganic ion transport and metabolism | 2.2 | 60.9 | 0.0 |
| bactNOG19139 | Methyltransferase type | [Q] Secondary metabolites biosynthesis, transport and catabolism | 3.2 | 69.6 | 17.4 |
| bactNOG00069 | Transporter protein | [R] General function prediction only | 27.0 | 95.7 | 43.5 |
| bactNOG28451 | Specifically catalyzes the dephosphorylation of 2- phosphoglycolate. Is involved in the dissimilation of the intracellular 2-phosphoglycolate formed during the DNA repair of 3'-phosphoglycolate ends, a major class of DNA lesions induced by oxidative stress (By similarity) protein | [R] General function prediction only | 13.2 | 95.7 | 30.4 |
| bactNOG07614 | Glutamine synthetase | [R] General function prediction only | 8.2 | 91.3 | 34.8 |
| bactNOG10082 | SAM dependent methyltransferase | [R] General function prediction only | 7.2 | 82.6 | 17.4 |
| bactNOG20523 | Sugar phosphatase; YidA; catalyzes the dephosphorylation of erythrose 4-phosphate (preferred substrate), mannose 1-phosphate and p-nitrophenyl phosphate; hydrolyzes the alpha-D-glucose-1-phosphate but not the beta form; member of the haloacid dehalogenase-like hydrolases superfamily and Cof family of proteins | [R] General function prediction only | 10.2 | 78.3 | 21.7 |
| bactNOG28216 | Hydrolase | [R] General function prediction only | 2.0 | 73.9 | 13.0 |
| bactNOG29366 | Trna (guanine-N(7)-)-methyltransferase | [R] General function prediction only | 6.0 | 69.6 | 13.0 |
| bactNOG33233 |  | [R] General function prediction only | 2.0 | 69.6 | 8.7 |
| bactNOG04076 | Zinc phosphodiesterase, which displays some tRNA 3'- processing endonuclease activity. involved in tRNA maturation, by removing a 3'-trailer from precursor tRNA (By similarity) | [R] General function prediction only | 5.1 | 65.2 | 4.3 |
| bactNOG31052 | Had-Superfamily hydrolase, subfamily IA, variant 3 | [R] General function prediction only | 16.0 | 65.2 | 13.0 |
| bactNOG50680 |  | [R] General function prediction only | 4.1 | 65.2 | 8.7 |
| bactNOG29488 | Transferase | [R] General function prediction only | 3.4 | 60.9 | 4.3 |
| bactNOG33333 | Cytidylate kinase | [S] Function unknown | 1.1 | 100.0 | 21.7 |
| bactNOG09783 |  | [S] Function unknown | 2.5 | 95.7 | 30.4 |
| bactNOG09927 |  | [S] Function unknown | 6.8 | 87.0 | 21.7 |
| bactNOG20334 |  | [S] Function unknown | 4.6 | 87.0 | 34.8 |
| bactNOG29149 |  | [S] Function unknown | 0.4 | 87.0 | 4.3 |
| bactNOG67809 |  | [S] Function unknown | 0.4 | 87.0 | 8.7 |
| bactNOG03627 |  | [S] Function unknown | 24.3 | 82.6 | 26.1 |
| bactNOG46203 |  | [S] Function unknown | 0.5 | 82.6 | 8.7 |
| bactNOG31698 | DNA primase | [S] Function unknown | 2.6 | 78.3 | 13.0 |
| bactNOG54851 |  | [S] Function unknown | 0.4 | 78.3 | 8.7 |
| bactNOG65906 |  | [S] Function unknown | 0.8 | 78.3 | 4.3 |
| bactNOG67837 |  | [S] Function unknown | 0.2 | 78.3 | 8.7 |
| bactNOG67840 |  | [S] Function unknown | 3.2 | 78.3 | 17.4 |
| bactNOG73501 |  | [S] Function unknown | 0.2 | 78.3 | 8.7 |
| bactNOG13083 |  | [S] Function unknown | 8.0 | 73.9 | 21.7 |
| bactNOG43319 | Adenylate cyclase | [S] Function unknown | 1.6 | 73.9 | 8.7 |
| bactNOG44258 | Protein involved in conjugation with cellular fusion | [S] Function unknown | 1.9 | 73.9 | 8.7 |
| bactNOG58688 |  | [S] Function unknown | 0.6 | 73.9 | 4.3 |
| bactNOG64392 | Stage II sporulation protein | [S] Function unknown | 0.4 | 73.9 | 17.4 |
| bactNOG78191 |  | [S] Function unknown | 0.2 | 73.9 | 4.3 |
| bactNOG84007 | Activator of cell division through the inhibition of ftsZ GTPase activity, therefore promoting ftsZ assembly into bundles of protofilaments necessary for the formation of the division Z ring. It is recruited early at mid-cell but it is not essential for cell division (By similarity) | [S] Function unknown | 0.6 | 73.9 | 21.7 |
| bactNOG19481 |  | [S] Function unknown | 3.7 | 69.6 | 13.0 |
| bactNOG19619 |  | [S] Function unknown | 1.2 | 69.6 | 8.7 |
| bactNOG41708 |  | [S] Function unknown | 4.5 | 69.6 | 8.7 |
| bactNOG60000 |  | [S] Function unknown | 3.5 | 69.6 | 17.4 |
| bactNOG61174 | Replication initiator protein | [S] Function unknown | 0.2 | 69.6 | 0.0 |
| bactNOG71139 |  | [S] Function unknown | 0.2 | 69.6 | 13.0 |
| bactNOG76652 |  | [S] Function unknown | 1.2 | 69.6 | 0.0 |
| bactNOG17369 |  | [S] Function unknown | 1.4 | 65.2 | 13.0 |
| bactNOG22679 |  | [S] Function unknown | 1.0 | 65.2 | 4.3 |
| bactNOG50416 | Branched-Chain amino acid transport protein | [S] Function unknown | 0.9 | 65.2 | 4.3 |
| bactNOG66939 |  | [S] Function unknown | 0.2 | 65.2 | 0.0 |
| bactNOG10331 |  | [S] Function unknown | 3.2 | 60.9 | 4.3 |
| bactNOG13347 |  | [S] Function unknown | 3.6 | 60.9 | 4.3 |
| bactNOG21494 |  | [S] Function unknown | 1.4 | 60.9 | 8.7 |
| bactNOG39396 |  | [S] Function unknown | 1.1 | 60.9 | 8.7 |
| bactNOG46011 |  | [S] Function unknown | 0.7 | 60.9 | 8.7 |
| bactNOG47345 |  | [S] Function unknown | 1.5 | 60.9 | 8.7 |
| bactNOG70843 |  | [S] Function unknown | 0.4 | 60.9 | 0.0 |
| bactNOG81954 |  | [S] Function unknown | 0.5 | 60.9 | 8.7 |
| bactNOG70972 | Sortase B | [S] Function unknown | 1.7 | 56.5 | 4.3 |
| bactNOG80674 |  | [S] Function unknown | 0.2 | 56.5 | 4.3 |
| bactNOG95081 |  | [S] Function unknown | 0.2 | 56.5 | 4.3 |
| bactNOG96197 |  | [S] Function unknown | 0.2 | 56.5 | 4.3 |
| bactNOG97104 |  | [S] Function unknown | 0.2 | 56.5 | 0.0 |
| bactNOG36304 |  | [S] Function unknown | 1.2 | 52.2 | 0.0 |
| bactNOG36880 |  | [S] Function unknown | 9.5 | 52.2 | 0.0 |
| bactNOG44818 | Heptaprenyl diphosphate synthase component I | [S] Function unknown | 0.6 | 52.2 | 0.0 |

bactNOGs are presented for which the number of gut genomes where the bactNOG is represented exceeds the number of non-gut genomes where the bactNOG is represented by at least 12. Abundance, % of species where the bactNOG is represented, in the eggNOG v.3.0 database, in 23 “gut” genomes (GUT), or in 23 “non-gut” genomes (NONGUT), respectively.
