# Supplementary material for: Functional Comparison of Bacteria from the Human Gut and Closely Related Non-Gut Bacteria Reveals the Importance of Conjugation and a Paucity of Motility and Chemotaxis Functions in the Gut Environment
Source: PLoS One. 2016 Jul 14;11(7):e0159030. doi: 10.1371/journal.pone.0159030 (PMC4945068; doi:10.1371/journal.pone.0159030)
Supplement: S2 Table — (DOCX) [file pone.0159030.s002.docx]

**S2 Table. BactNOGs underrepresented in gut bacteria.**

| **eggNOG** | **Function** | **Functional category** | **Abundance, %** | | |
| --- | --- | --- | --- | --- | --- |
|  |  |  | **egg**  **NOG v.3.0** | **GUT** | **NON**  **GUT** |
| bactNOG01470 | Cell division protein FtsA | [D] Cell cycle control, cell division, chromosome partitioning | 65.1 | 8.7 | 65.2 |
| bactNOG00525 | Utp-Glucose-1-Phosphate uridylyltransferase | [M] Cell wall/membrane/envelope biogenesis | 63.7 | 30.4 | 82.6 |
| bactNOG01018 | Udp-N-Acetylglucosamine 2-epimerase | [M] Cell wall/membrane/envelope biogenesis | 49.1 | 34.8 | 95.7 |
| bactNOG01716 | Undecaprenyl-Phosphate alpha-N protein | [M] Cell wall/membrane/envelope biogenesis | 41.4 | 17.4 | 69.6 |
| bactNOG05792 | Cyanophycin synthetase | [M] Cell wall/membrane/envelope biogenesis | 18.5 | 0.0 | 56.5 |
| bactNOG00716 | Flagellin protein | [N] Cell motility | 38.8 | 17.4 | 82.6 |
| bactNOG01465 | Flagellar hook protein FlgE | [N] Cell motility | 41.7 | 13.0 | 65.2 |
| bactNOG01751 | Flagellar biosynthesis protein FlhB; membrane protein responsible for substrate specificity switching from rod/hook-type export to filament-type export | [N] Cell motility | 37.9 | 13.0 | 82.6 |
| bactNOG02127 | Flagellar biosynthesis protein FliP; FliP, with proteins FliQ and FliR, forms the core of the central channel in the flagella export apparatus | [N] Cell motility | 42.3 | 21.7 | 82.6 |
| bactNOG02318 | Flagellar motor switch protein FliM | [N] Cell motility | 41.9 | 21.7 | 78.3 |
| bactNOG02345 | Flagellar motor switch protein | [N] Cell motility | 41.0 | 21.7 | 82.6 |
| bactNOG02669 | Flagellar biosynthesis protein FlhA | [N] Cell motility | 43.8 | 21.7 | 82.6 |
| bactNOG04544 | Flagellar basal body rod protein FlgG | [N] Cell motility | 41.4 | 21.7 | 82.6 |
| bactNOG16417 | Flagellar motor protein MotD; Homologous to MotB. These organism have both MotB and MotD. With MotC (a MotA homolog) forms the ion channels that couple flagellar rotation to proton/sodium motive force across the membrane and forms the stator elements of the rotary flagellar machine. Either MotAB or MotCD is sufficient for swimming, but both are necessary for swarming motility | [N] Cell motility | 13.8 | 4.3 | 56.5 |
| bactNOG30371 | Flagellar basal body rod protein | [N] Cell motility | 21.8 | 21.7 | 82.6 |
| bactNOG43795 | Protein FliQ | [N] Cell motility | 30.4 | 17.4 | 82.6 |
| bactNOG43852 | Flagellar protein FliS | [N] Cell motility | 16.5 | 21.7 | 78.3 |
| bactNOG44208 | Flagellar motor switch protein | [N] Cell motility | 17.6 | 8.7 | 60.9 |
| bactNOG44267 | Flagellar hook capping protein | [N] Cell motility | 9.9 | 8.7 | 65.2 |
| bactNOG47686 |  | [N] Cell motility | 3.8 | 0.0 | 52.2 |
| bactNOG12090 | Methyltransferase, CheR | [N] Cell motility/ [T] Signal transduction mechanisms | 5.0 | 4.3 | 65.2 |
| bactNOG27823 | Chec, inhibitor of MCP methylation protein | [N] Cell motility/ [T] Signal transduction mechanisms | 3.6 | 4.3 | 56.5 |
| bactNOG00811 | Atp-Dependent protease | [O] Posttranslational modification, protein turnover, chaperones/[U] Intracellular trafficking, secretion, and vesicular transport | 26.0 | 4.3 | 56.5 |
| bactNOG04772 | Peptidase M16 | [O] Posttranslational modification, protein turnover, chaperones/[U] Intracellular trafficking, secretion, and vesicular transport | 35.8 | 13.0 | 73.9 |
| bactNOG16773 | Atp-Dependent protease | [O] Posttranslational modification, protein turnover, chaperones/[U] Intracellular trafficking, secretion, and vesicular transport | 6.5 | 0.0 | 56.5 |
| bactNOG26624 | Protein involved in cell division | [O] Posttranslational modification, protein turnover, chaperones/[U] Intracellular trafficking, secretion, and vesicular transport | 5.5 | 0.0 | 52.2 |
| bactNOG00287 | Involved in the modulation of the chemotaxis system; catalyzes the demethylation of specific methylglutamate residues introduced into the chemoreceptors (methyl-accepting chemotaxis proteins) by cheR | [T] Signal transduction mechanisms | 39.0 | 13.0 | 82.6 |
| bactNOG00749 | Methyl-Accepting chemotaxis protein | [T] Signal transduction mechanisms | 49.1 | 21.7 | 73.9 |
| bactNOG01870 | Protein involved in chemotaxis | [T] Signal transduction mechanisms | 46.3 | 17.4 | 82.6 |
| bactNOG22977 | Histidine kinase | [T] Signal transduction mechanisms | 8.8 | 13.0 | 73.9 |
| bactNOG23272 | Regulator protein | [T] Signal transduction mechanisms | 15.4 | 4.3 | 56.5 |
| bactNOG23776 | Diguanylate cyclase | [T] Signal transduction mechanisms | 157.0 | 0.0 | 65.2 |
| bactNOG28166 | Response regulator receiver protein | [T] Signal transduction mechanisms | 9.5 | 21.7 | 78.3 |
| bactNOG35800 | Methyl-Accepting chemotaxis protein | [T] Signal transduction mechanisms | 2.3 | 0.0 | 52.2 |
| bactNOG03842 | Abc-Transporter protein | [V] Defense mechanisms | 20.6 | 4.3 | 65.2 |
| bactNOG00070 | Catalyzes the attachment of glutamate to tRNA in a two-step reaction: glutamate is first activated by ATP to form Glu-AMP and then transferred to the acceptor end of tRNA protein | [J] Translation, ribosomal structure and biogenesis | 80.6 | 8.7 | 60.9 |
| bactNOG04795 |  | [J] Translation, ribosomal structure and biogenesis | 8.7 | 13.0 | 65.2 |
| bactNOG15344 | Peptide deformylase | [J] Translation, ribosomal structure and biogenesis | 8.3 | 0.0 | 52.2 |
| bactNOG43731 | 50S ribosomal protein L34; in Escherichia coli transcription of this gene is enhanced by polyamines | [J] Translation, ribosomal structure and biogenesis | 63.9 | 34.8 | 95.7 |
| bactNOG01662 | Transcriptional regulator, GntR family protein | [K] Transcription | 38.7 | 30.4 | 87.0 |
| bactNOG12540 | Regulator protein | [K] Transcription | 10.2 | 4.3 | 69.6 |
| bactNOG16918 | Pur operon repressor protein | [K] Transcription | 15.2 | 4.3 | 91.3 |
| bactNOG20038 | Cell envelope-related transcriptional attenuator; TIGRFAM: cell envelope-related function transcriptional attenuator, LytR/CpsA family; PFAM: cell envelope-related transcriptional attenuator protein | [K] Transcription | 12.7 | 21.7 | 87.0 |
| bactNOG31290 | Ferric uptake regulator protein | [K] Transcription | 9.2 | 8.7 | 60.9 |
| bactNOG46911 | Transcriptional regulator, CopG family protein | [K] Transcription | 6.3 | 4.3 | 60.9 |
| bactNOG00059 | Dehydrogenase | [C] Energy production and conversion | 68.3 | 17.4 | 78.3 |
| bactNOG02069 | Flagellum-Specific ATP synthase | [C] Energy production and conversion | 43.2 | 21.7 | 82.6 |
| bactNOG00957 | Catalyzes the condensation of the acetyl group of acetyl-CoA with 3-methyl-2-oxobutanoate (2-oxoisovalerate) to form 3-carboxy-3-hydroxy-4-methylpentanoate protein | [E] Amino acid transport and metabolism | 49.1 | 4.3 | 65.2 |
| bactNOG01885 | Aspartate-Semialdehyde dehydrogenase | [E] Amino acid transport and metabolism | 61.3 | 17.4 | 82.6 |
| bactNOG03292 | Amino acid protein | [E] Amino acid transport and metabolism | 35.4 | 8.7 | 60.9 |
| bactNOG09186 | Amino acid protein | [E] Amino acid transport and metabolism | 16.8 | 0.0 | 60.9 |
| bactNOG12565 | Atp:Guanido phosphotransferase | [E] Amino acid transport and metabolism | 12.7 | 21.7 | 73.9 |
| bactNOG00229 | Phosphoribosylaminoimidazolecarboxamide formyltransferase/IMP cyclohydrolase | [F] Nucleotide transport and metabolism | 75.2 | 13.0 | 95.7 |
| bactNOG00965 | Phosphoribosylaminoimidazole carboxylase ATPase subunit | [F] Nucleotide transport and metabolism | 53.4 | 0.0 | 60.9 |
| bactNOG01343 | Plays an important role in the de novo pathway of purine nucleotide biosynthesis protein | [F] Nucleotide transport and metabolism | 76.7 | 30.4 | 87.0 |
| bactNOG02636 | Ribose-Phosphate pyrophosphokinase | [F] Nucleotide transport and metabolism | 79.3 | 30.4 | 95.7 |
| bactNOG18657 | Bifunctional pyrimidine regulatory protein PyrR uracil phosphoribosyltransferase; regulates pyrimidine biosynthesis by binding to the mRNA of the pyr genes, also has been shown to have uracil phosphoribosyltransferase activity | [F] Nucleotide transport and metabolism | 35.0 | 17.4 | 69.6 |
| bactNOG23297 | Thymidylate kinase | [F] Nucleotide transport and metabolism | 73.1 | 13.0 | 65.2 |
| bactNOG25456 | Glutamine amidotransferase, subunit PdxT; with PdxST is involved in the biosynthesis of pyridoxal 5'-phosphate; PdxT catalyzes the hydrolysis of glutamine to glutamate and ammonia; PdxS utilizes the ammonia to synthesize pyridoxal 5'-phosphate | [H] Coenzyme transport and metabolism | 17.4 | 4.3 | 65.2 |
| bactNOG00578 | Drug resistance transporter, EmrB/QacA protein | [P] Inorganic ion transport and metabolism | 56.8 | 26.1 | 78.3 |
| bactNOG00626 | Arsenical-Resistance protein | [P] Inorganic ion transport and metabolism | 29.8 | 8.7 | 60.9 |
| bactNOG00453 | Metal dependent phosphohydrolase | [R] General function prediction only | 18.8 | 4.3 | 65.2 |
| bactNOG04713 |  | [R] General function prediction only | 17.1 | 13.0 | 73.9 |
| bactNOG11597 | Ribonuclease Z; member of metallo-beta-lactamase family; the purified enzyme from Escherichia coli forms dimeric zinc phosphodiesterase; in Bacillus subtilis this protein is a 3'-tRNA processing endoribonuclease and is essential while in Escherichia coli it is not; associates with two zinc ions | [R] General function prediction only | 19.9 | 4.3 | 56.5 |
| bactNOG43940 | Protein involved in protein secretion | [R] General function prediction only | 17.7 | 21.7 | 73.9 |
| bactNOG02831 |  | [S] Function unknown | 24.4 | 17.4 | 82.6 |
| bactNOG12472 | Alkaline phosphatase | [S] Function unknown | 21.8 | 13.0 | 69.6 |
| bactNOG37372 | Stage II sporulation protein M | [S] Function unknown | 6.6 | 4.3 | 60.9 |
| bactNOG48219 |  | [S] Function unknown | 2.6 | 0.0 | 60.9 |
| bactNOG49295 |  | [S] Function unknown | 6.8 | 4.3 | 65.2 |
| bactNOG52894 |  | [S] Function unknown | 16.0 | 4.3 | 56.5 |

bactNOGs are presented for which the number of non-gut genomes where the bactNOG is represented exceeds the number of gut genomes where the bactNOG is represented by at least 12. Abundance, % of species where the bactNOG is represented, in the eggNOG v.3.0 database, in 23 “gut” genomes (GUT), or in 23 “non-gut” genomes (NONGUT), respectively.
